# Coenzyme Q9 Derived from *Oudemansiella raphanipes* Attenuates Mucin Biosynthesis Induced by *Pseudomonas aeruginosa* Pyocyanin in Human Bronchial Epithelial Cells

**DOI:** 10.3390/antiox15101225

**Published:** 2026-09-23

**Authors:** Gun-Do Kim, Min-Jae Kang, Keun Ki Kim, Woosuk Choi, Gee W. Lau

**Affiliations:** 1 Department of Microbiology, College of Natural Science, Pukyong National University, Busan 48513, Republic of Korea; gundokim@pknu.ac.kr (G.-D.K.);; 2 Department of Life Science and Environmental Biochemistry, College of Natural Resource and Life Sciences, Pusan National University, Miryang 50463, Republic of Korea; 3 Department of Pathobiology, College of Veterinary Medicine, University of Illinois at Urbana-Champaign, Urbana, IL 61802, USA

**Keywords:** *Pseudomonas aeruginosa* pyocyanin, oxidative stress, mucus hypersecretion, *Oudemansiella raphanipes*, Coenzyme Q9, mitophagy, mitochondrial biogenesis

## Abstract

Pyocyanin is a potent redox-cycling secondary metabolite secreted by the respiratory pathogen *Pseudomonas aeruginosa*. During chronic *P. aeruginosa* infections, pyocyanin induces persistent oxidative stress in airway epithelial cells, ultimately leading to goblet cell hyperplasia and metaplasia alongside mucus hypersecretion. Because PCN triggers a redox imbalance by rapid depletion of host antioxidants, restoring cellular antioxidative capacity is essential to mitigate its pathophysiology. Herein, we demonstrate that Coenzyme Q9 (CoQ9) from the edible mushroom *Oudemansiella raphanipes* attenuates pyocyanin-induced mucus hypersecretion in primary human bronchial epithelial cells by restoring mitochondrial integrity to alleviate oxidative stress. CoQ9 pretreatment attenuates MUC5AC and MUC5B mucin expression by inactivating mucin-promoting signaling cascades, including the EGFR-AKT/ERK1/2 and STAT6-SPDEF axes. In addition to its reactive oxygen species scavenging properties, CoQ9 significantly downregulated pyocyanin-induced mitochondrial superoxide generation by restoring the SIRT3-SOD2 axis. Specifically, CoQ9 pretreatment improved mitochondrial integrity by preserving PINK1-mediated mitophagy and enhancing mitochondrial biogenesis through activation of the AMPK-PGC1α axis, thereby restoring ATP levels under PCN exposure.

## 1. Introduction

Mucus hypersecretion is a common pathological feature in patients with chronic muco-obstructive diseases, including chronic obstructive pulmonary disease (COPD), cystic fibrosis (CF), and non-CF bronchiectasis, and chronic bronchitis [1,2,3,4,5]. Despite the protective roles of airway mucus against inhaled toxicants, pollutants, and microbial infection [6], paradoxically, excessive mucus production and failure in mucus clearance cause airway obstruction and create a pathological microenvironment that facilitates chronic microbial colonization, leading to increased patient morbidity and mortality [7,8,9,10]. Thus, restoring airway mucus homeostasis is essential to improving the quality of life for these patients.

Airway mucus homeostasis is tightly regulated by the balance between mucociliary clearance and mucus production [11]. Mucin glycoproteins are the major components of mucus that determine its volume, viscoelasticity, and hydration status [12]. MUC5AC and MUC5B are the predominant secretory mucins of airway mucus in both healthy and diseased lungs, which are mainly produced by airway goblet cells and submucosal gland mucous cells, respectively [13]. In diseased airways, the hyperproduction of MUC5AC and MUC5B significantly elevates mucus thickness and viscosity, thereby impeding mucociliary clearance [14]. Despite extensive research into therapeutic strategies for alleviating mucin hyperproduction in patients with muco-obstructive diseases, efficacious treatment options remain limited.

Airway goblet cell development and mucin biosynthesis are tightly regulated by Forkhead Box A2 (FOXA2), a major transcriptional factor that maintains airway mucus homeostasis [15,16,17]. In unstimulated airway epithelial basal and club cells, FOXA2 represses the transcription of *MUC5AC* and *MUC5B* genes by sequestering promoter regions of both genes. However, when the airway epithelial cells are exposed to stressed conditions, including microbial infections or allergens that trigger inflammation and oxidative stress, FOXA2 is inactivated and depleted, allowing transdifferentiation of basal and club cells into mucin-secreting goblet cells as well as *MUC5AC* and *MUC5B* overexpression [18,19]. The activity and stability of FOXA2 are regulated by two signaling pathways, including epidermal growth factor receptor (EGFR)-protein kinase B (AKT)/extracellular signaling regulated kinase-1/2 (ERK1/2), as well as the interleukin-4/ interleukin-13 receptor (IL-4/IL-13R)-signal transducer and activator of transcription 6 (STAT6)—SAM pointed domain-containing Ets transcription factor (SPDEF) axes [19,20,21,22]. While EGFR-mediated signaling reduces nuclear FOXA2 abundance by AKT-mediated phosphorylation and subsequent cytoplasmic sequestration and proteasomal degradation [19,20,23,24,25], IL-4/IL-13R-mediated signaling downregulates basal FOXA2 levels by suppressing its transcription through the induction of SPDEF [18,26,27]. Based on these molecular networks governing FOXA2 turnover, previous studies have demonstrated that pharmacotherapeutic approaches aimed at increasing FOXA2 levels effectively reverse airway mucin overproduction and goblet cell hyperplasia [19,20]. These findings suggest that restoring FOXA2 expression could be a viable approach for reestablishing airway mucus homeostasis in diseased airways.

*Pseudomonas aeruginosa* (*PA*) is an opportunistic pathogen notoriously recalcitrant to antibiotics and is recognized as a major pathogen that causes chronic pulmonary infections in patients with CF, non-CF bronchiectasis, COPD, primary ciliary dyskinesia (PCD), and ventilator-associated pneumonia (VAP), thereby accelerating the decline of lung function and increasing the recurrence of acute exacerbations [28,29,30,31]. A large repertoire of virulence factors allows *PA* to evade the host defense system and establish chronic infection [32,33]. Among virulence factors, pyocyanin (PCN) is a potent redox-cycling secondary metabolite that enables *PA* to kill competing microbiota and host cells as well as disrupting the host cellular defense system by generating excessive amounts of reactive oxygen species (ROS) and reactive nitrogen species (RNS) [34,35,36,37,38,39]. Specifically, PCN induces goblet cell hyperplasia and metaplasia alongside mucus hypersecretion through oxidative stress-induced hyperactivation of EGFR- and STAT6-mediated signaling pathways, which converge to downregulate FOXA2 function and expression [19,20,22,25,40,41,42]. Although the host antioxidant defense systems detoxify ROS and RNS [43,44], PCN disrupts this balance by rapidly depleting intracellular antioxidants and suppressing the expression and activity of antioxidant enzymes [25,43,45,46], thereby inducing a wider dysfunction, especially in major organelles such as mitochondria [34,47,48,49].

Mitochondria are essential organelles that maintain cell vitality by participating in the biosynthesis of the critical antioxidant CoQ10 as well as harboring large amounts of glutathione (GSH), which are vital for maintaining redox homeostasis, oxidative phosphorylation, and mitochondrial membrane potential [50,51]. The loss of antioxidants by persistent PCN redox cycling damages mitochondria by increasing the mitochondrial superoxide levels [45], causing functional abnormalities in host cells. The mitochondrial antioxidant defense system is tightly regulated by Sirtuin 3 (SIRT3), a mitochondria-specific NAD^+^-dependent histone deacetylase [52]. In addition to regulatory roles in mitochondrial metabolism, SIRT3 maintains mitochondrial redox homeostasis through the activation and expression of mitochondrial antioxidant enzymes such as superoxide dismutase 2 (SOD2) [53,54,55] and glutathione peroxidase 4 (GPX4) [56]. Relevant to the pathogenesis of mucin biosynthesis, the depletion of GPX4 [56] and SIRT3 [57] induces mucin hyperproduction, whereas SIRT3 activation reverses this pathological response. Accordingly, restoring mitochondrial redox homeostasis represents a potential therapeutic strategy to ameliorate oxidative stress-mediated mucin production and goblet cell transdifferentiation. Despite the protective effects of CoQ9 against oxidative stress-induced damage, its roles in regulating mitochondrial integrity and mucin biosynthesis are largely unknown.

Medicinal herbs have emerged as promising bioresources for the development of alternative therapeutics against diverse human diseases. The mushroom *Oudemansiella raphanipes* is widely utilized as a food ingredient due to its rich nutritive value and diverse array of bioactive compounds [58]. It has been reported that *O. raphanipes* extracts possess antioxidant properties [59,60,61]. However, the specific bioactive components responsible for these antioxidant effects remain unknown. Coenzyme Q9 (CoQ9), a member of the ubiquinone family essential for respiratory chain function [62,63], is abundant in organisms ranging from yeasts to rodents, rather than in humans [64,65]. Previous studies have demonstrated the physiological roles of CoQ9 in protecting against cardiovascular diseases and encephalomyopathy by stabilizing mitochondrial function and scavenging free radicals [65,66,67,68]. In addition, exogenous supplementation of CoQ9 ameliorates oxidative stress by mitigating free radical-induced injury in hepatocytes [69], suggesting that CoQ9 may reverse oxidative stress-mediated cellular damage.

In this study, we demonstrated that CoQ9 isolated from *O. raphanipes* suppresses mucin hypersecretion and goblet cell differentiation in PCN-exposed primary human bronchial epithelial cells by rescuing damaged mitochondrial functions. Mechanistically, CoQ9 prevents the downregulation of FOXA2 by attenuating the hyperactivation of the EGFR-AKT/ERK1/2 and STAT6-SPDEF. In addition, CoQ9 diminishes mitochondrial oxidative stress by restoring the SIRT3-SOD2 expression and preserving mitochondrial integrity by promoting PINK1-mediated mitophagy and mitochondrial biogenesis via the AMPK-PGC1α, thereby minimizing PCN-induced ATP depletion.

## 2. Materials and Methods

### 2.1. Chemicals and Antibodies

PCN (#R9532), WST-1 reagent (#5015944001), and DAPI (#10236276001) were purchased from Sigma-Aldrich (St. Louis, MO, USA). Primary antibodies: anti-MUC5AC (#SC-20118), anti-MUC5B (#SC-20119), anti-FOXA2 (#SC-101060), anti-EGFR (#SC-03), anti-p-EGFR (#SC-101668), anti-p-STAT6 (#SC-71793), anti-AKT (#SC-8312), anti-p-AKT (#SC-16646), anti-PGC1α (#SC-517380), anti-SOD2 (#SC-137254), and anti-GAPDH (#SC-166545) were purchased from Santa Cruz Biotechnology (Dallas, TX, USA); anti-STAT6 (#9362), anti-p-AMPK (#2535), anti-AMPK (#2793), anti-SIRT3 (#2627), anti-TOM20 (#42406), anti-LC3B (#83506), and anti-Histone H3 (#9715) were purchased from Cell Signaling Technology (Danvers, MA, USA); anti-PINK1 (#23274-1-AP, Proteintech, Rosemont, IL, USA), anti-p-PGC1α (#AF6650, R & D systems, Minneapolis, MN, USA), anti-Acetyl-SOD2 (#PA5-114697, Invitrogen, Waltham, MA, USA), and Anti-NRF2 (Ab62352, Abcam, Waltham, MA, USA). Secondary antibodies: anti-rabbit IgG, HRP-linked antibody (7074S, Cell Signaling Technology), and anti-mouse IgG, HRP-linked antibody (7076S, Cell Signaling Technology) were utilized for Western blot analysis. Anti-rabbit, Alexa Fluor 488 (#A11008, Invitrogen) and anti-mouse, Alexa Fluor 594 (#A11004, Invitrogen) were used for immunofluorescence staining.

### 2.2. Extraction of Organic Fraction from O. raphanipes

*O. raphanipes* dried powder (80g) was extracted by adding organic solvents and water, 10 times the weight of the dried powder. Organic fractions were extracted sequentially by using hexane, chloroform (CHCl3), and methanol (Figure 1A). Each extract was concentrated with a rotary vacuum evaporator (EYELA, Tokyo, Japan).

### 2.3. Chromatography

Among the solvent extracts, CHCl_3_ extracts were used for material separation. The first separation by NP-silica gel column chromatography resulted in 78 fractions. The elution solvent was a mixture of hexane and ethyl acetate and was fractionated by increasing the polarity. The fraction was divided into nine groups of similar R*f* values using thin layer chromatography (TLC). The anti-inflammatory substances were carried out twice with TLC (1 mm and 0.25 mm, respectively). The substances separated by TLC were used for GC/MS analysis to confirm the degree of separation of the material.

### 2.4. Measurement of Spectra

UV/visible spectrophotometer measurement and 1D-NMR (^1^H-NMR, ^13^C-NMR) and 2D-NMR (gCOSY, gHSQC, gHMBC) analyses were performed for structural identification of the separated material. FAB-MS analysis was performed to measure the molecular weight.

### 2.5. Extraction and Purification

Among the *O. raphanipes* fractions, the chloroform extracted fraction showed the highest antioxidant activity in regulating nitrite production by the Griess assay. The CHCl_3_ extracts yielded 1.75 g compound from 80 g of mushrooms. The CHCl_3_ extracts at 100 μg/mL inhibited nitrate production by 62% compared to the control treated with lipopolysaccharides (LPS), and there was no cytotoxicity. CHCl_3_ extracts were used to purify the anti-inflammatory substance by column chromatography, followed by two times with thin layer chromatography (TLC). The anti-inflammatory activity was investigated at each separation step, and a highly active fraction was used in the next stage. NP-TLC (Figure 1B,C) and FID-GC (Figure 1D) were performed to confirm the purity of the isolated anti-inflammatory active substance. No impurities were identified.

### 2.6. Structure Identification

The structural identification of the active antioxidant substance isolated from *O. raphanipes* was determined by various instrumental analyses. The UV/vis spectrophotometer measurement revealed maximum absorption at 275 nm, and peaks were also observed at 250 nm, confirming that there was a double bond in the structure. From the ^1^H-NMR assignment, isoprenoid methyl groups δ 1.59 ppm, terminal methyl δ 1.67 ppm, isoprenoid methylene(-CH_2_) δ 2.09 to δ 2.04 ppm, methyl on C-2 δ 2.01 ppm, methoxyl protons(-OCH_3_) δ 3.99 ppm, isopreneid (isoprene) signals were identified, which show the same pattern as CoQ10 NMR spectrum as reported by Ravada et al. [70,71,72]. The value of ^13^C-NMR chemical shift was found to be the carbonyl carbon (δ 184.73 and 183.88 ppm) of the benzene ring, methoxyl carbones (δ 17.66 and 16.33 ppm), and methyl carbon of the C2 (δ 11.95 ppm] signal. The carbon –C, -CH signal of the isoprene structure was observed at δ 137.61 ppm and 124.38 ppm, respectively, while the -CH_2_ signal of isoprene was observed at δ 39.72 ppm and 27.18 ppm. The methyl group of vinyl was confirmed at δ 16.00 ppm. These are the general analysis values of the isoprenoid structure which can be easily confirmed in structures such as Vitamin A and squalene. ^13^C-NMR assignment values and patterns were analyzed based on the previous study [72]. It was similar to the reported CoQ10 NMR shift values [70,72]. As a result of FAB-MS analysis, a molecular peak was confirmed at *m*/*z* 796.8 (M+1). Based on NMR and MS data, purified antioxidant substances were identified as CoQ 9 (C_54_H_82_O_4_).

### 2.7. Cell Culture

Primary human bronchial epithelial cells (NHBE) (#CC-2540) were obtained from Lonza (Portsmouth, NH, USA). NHBE cells were cultured in submerged and air–liquid interface (ALI) conditions at 37 °C in 5% CO_2_. For submerged culture, the cells were maintained in the bronchial epithelial growth media (BEGM) supplemented with SingleQuots kits from Lonza. At Passage 3, the cells were trypsinized and seeded onto 12-well plates containing coverslips for immunofluorescence analysis and 96-well plates for ROS detection assays, respectively. For ALI culture, 0.5 × 10^4^ NHBE cells were seeded onto Costar transwell inserts with 0.4 μm pore size (CLS3381, Corning, Kennebunk, ME, USA ) and cultured in submerged condition for 5 days by adding BEGM in both apical and basolateral chambers. On day 6, the media of both chambers were aspirated, and the Pneumo-cult ALI media (#05003, Stemcell Techonologies, Vancouver, BC, Canada) containing PneumoCult-ALI supplement (#05003), heparin (#07890), and hydrocortisone (#07925) was added into the basolateral chamber only. The cells were differentiated for 21 days before exposure to PCN with or without COQ9 for 6–24 h.

### 2.8. Cell Viability Assay

NHBE cells (1.0 × 10^4^) were seeded on 96-well plates and treated with PCN with or without CoQ9. After treatment, the cells were incubated with WST-1 reagent, diluted in the culture media at 1:10, for 3 h. The conditioned media were measured at 440 nm wavelength with a microtiter plate reader.

### 2.9. Total, Cytosolic and Nuclear Protein Extraction

NHBE cells grown in submerged culture were used to isolate total and nuclear proteins. For total protein extraction, the cells were lysed by RIPA buffer (150 mM NaCl, 1% Triton-X, 0.5% sodium deoxycholate, 0.1% SDS, 25 mM Tris-HCl (50 mM, pH 7.4) and incubated on ice for 1 h, with intermittent vortexing every 10 min. The lysates were centrifuged at 14,200 rpm for 20 min, and the supernatants were collected for Western blot analysis. Nuclear proteins were extracted by using the Nuclear & Cytoplasmic Extraction kit (#786-182, G-biosciences, St. Louis, MO, USA). Briefly, the cultured NHBE cells were incubated on ice for 10 min in the cytosolic lysis buffer and then centrifuged at 14,200 rpm for 10 min. The supernatant was collected as the cytosolic fraction. The pellets were resuspended in nuclear lysis buffer. The lysates were sonicated and subsequently incubated on ice for 1 h, with intermittent vortexing every 15 min. Finally, the lysates were centrifuged at 14,200 rpm for 20 min, and the supernatant was collected as the nuclear fraction. Protein quantity was assessed by the BCA Protein Assay kit (#23227, Thermo Scientific, Rockford, IL, USA).

### 2.10. Western Blot Analysis

Total and nuclear proteins were mixed with 2X sample loading buffer and were boiled at 100 °C for 5 min. The protein samples were separated on 10% SDS-PAGE gel and transferred to a nitrocellulose membrane (#88018, Thermo Fisher Scientific) for 3 h. The membranes were blocked in 5% skim milk in TBST for 30 min and then incubated with primary antibodies (diluted at 1:1000 in 5% BSA-TBST) overnight at 4 °C. On day 2, the membranes were incubated with HRP-conjugated anti-rabbit or anti-mouse IgG secondary antibody for 1 h at room temperature. After incubation, the immune complexes were visualized by the SuperSignal West Femto Maximum Sensitivity Substrate (#24096, Thermo Scientific). Semi-quantification of Western blots was performed using ImageJ (Ver.1.54g; JAVA 1.8.0_345). Signal intensity for each target protein was quantified and normalized to its corresponding loading control. Data from three independent experiments were calculated and presented as mean ± s.e.m., with statistical significance indicated.

### 2.11. Quantitative Real-Time Polymerase Chain Reaction (qRT-PCR)

NHBE cells cultured in submerged and ALI conditions were pretreated with CoQ9 for 1 h before exposure to PCN for 24 h, both at the indicated concentrations. After incubation, the cells were lysed by Trizol reagent (15596018, Ambion, Austin, Tx, USA). The lysates were mixed with 100% ethanol at a 1:1 ratio and centrifuged at 12,000× *g* for 15 min. The supernatant was collected and used for total RNA purification by using the Direct-zol RNA miniprep (R2061, Zymogen, Irvine, CA, USA). The quality of the purified total RNA was assessed using a Thermo Scientific Nanodrop 1000 spectrophotometer by measuring A_260_/A_280_ and A_260_/A_230_ absorbance ratios. Only RNA samples with both ratios approximating 2.0 were selected for downstream assays. The purified total RNA was converted to cDNA by using the Superscript III First-strand Synthesis System (#18080051, Invitrogen). The synthesized cDNAs were used to examine relative mRNA levels of *MUC5AC*, *MUC5B*, *FOXA2*, *SIRT3*, and *SOD2* normalized to *GAPDH* by qRT-PCR using Power SYBR green PCR Master Mix (#4367659, Applied Biosystems). The following primers were used. *GAPDH* sense (5′-TGGGCTACACTGAGCACCAG-3′), *GAPDH* anti-sense (5′-GGGTGTCGCTGTTGAAGTCA-3′), *MUC5AC* sense (5′-GGAACTGTGGGGACAGCTCTT-3′), *MUC5AC* anti-sense (5′-GTCACATTCCTCAGCGAGGTC-3′); *MUC5B* sense (5′-GTGAGGAGGACTCCTGTCAAGT-3′), *MUC5B* anti-sense (5′-CCTCGCAGAAGGTGATGTTG-3′); *FOXA2* sense (5′-AAGTGGGGGTCGAGACTTTG-3′), *FOXA2* anti-sense (5′-TGAACAACAGCAATGGAG-3′); *SIRT3* sense (5′-CCCTGGAAACTACAAGCCCAAC-3′), *SIRT3* anti-sense (5′-GCAGAGGCAAAGGTTCCATGAG-3′); *SOD2* sense (5′-CTGGACAAACCTCAGCCCTAAC-3′), *SOD2* anti-sense (5′-AACCTGAGCCTTGGACACCAAC-3′); *GCLC* sense (5′-GGGAGTGATTTCTGCATCTGTAGAT-3′), *GCLC* anti-sense (5′-TGAGTCATATCGGGATTTACTGATCCT-3′); *HO-1* sense (5′-CCAGGCAGAGAATGCTGAGTTC-3′), and *HO-1* anti-sense (5′-AAGACTGGGCTCTCCTTGTTGC-3′).

### 2.12. Immunofluorescence Analysis

NHBE cells cultured in submerged and ALI conditions were fixed with 4% paraformaldehyde for 15 min. After fixation, the cells were permeabilized with 0.5% Triton-X in TBS for 15 min and nuclear-stained with DAPI for 15 min. The cells were subsequently incubated for 1 h with 5% donkey serum in TBST blocking solution (D9663-10 mL, Sigma-Aldrich). The blocked cells were stained with primary antibodies, diluted at 1:100 in the blocking solution, overnight. On day 2, the primary antibodies were probed with Alexa Fluor 488-conjugated anti-rabbit IgG and Alexa Fluor 568-conjugated anti-mouse IgG secondary antibodies, diluted at 1:250 in the blocking solution, for 1 h. Fluorescence-labeled images were captured with a Nikon A1R confocal microscope.

### 2.13. DPPH Assay

2,2-Diphenyl-1-picrylhydrazyl (DPPH) was purchased from Sigma Aldrich (#D9132). For the DPPH assay, CoQ9 was dissolved in dimethyl sulfoxide (DMSO) to obtain a 10 mM stock solution. The DPPH radical solution was prepared at the 120 μM concentration with 95% ethanol. A total of 10 μL of diluted CoQ9 (in 50, 75, and 100 μM) were added into a 96-well plate and mixed with 190 μL DPPH solution. The mixtures were incubated at room temperature for 30 min in the dark. After the reaction, absorbance was measured at 517 nm by using a microplate reader. The absorbance values were used to calculate the free radical scavenging activity (RSA) by following the formula: RSA (%) = [(blank − sample)/blank] × 100. The absorbance value represents the mean of triplicate samples.

### 2.14. ROS Quantification Assay

Total intracellular ROS levels were measured by the Oxiselect^TM^ in vitro ROS/RNS assay kit (#STA-347, Cell Biolabs, Inc., San Diego, CA, USA) following manufacturer’s instruction.

### 2.15. Mitochondrial Superoxide Generation Detection

To detect mitochondrial superoxide, MitoBright ROS Deep Red mitochondrial superoxide detection kit (#MT16-10, Dojindo Laboratories, Kumamoto, Japan) was utilized following the manufacturer’s recommendation. Briefly, 1.0 × 10^4^ NHBE cells were seeded in µ-slide 8-well plates (#80821, Ibidi, Grafelfing, Bayern, Germany). The cells were pretreated for 1 h with 100 µM CoQ9 before exposure to pyocyanin (5 µg/mL) for 6 h. After the treatment, the conditioned media were aspirated, and the cells were incubated with 200 µL ROS Deep Red working solution (#MT16-10, Dojindo Laboratories) for 30 min at 37 °C in 5% CO_2_. Fluorescence signals were visualized with a Nikon A1R confocal microscope. Fluorescence intensities were measured by ImageJ.

### 2.16. Intracellular ATP Quantification

Changes in ATP levels within NHBE cells treated with PCN and CoQ were assessed by the ATP assay kit (#ab83355, Abcam) according to the manufacturer’s instruction.

### 2.17. Glycolysis and Mitochondrial Membrane Potential Measurement

Mitochondrial integrity-dependent metabolic rewiring was measured by Glycolysis/JC-1 MitoMP assay kit (#G272-10, Dojindo) following the manufacturer’s instruction.

### 2.18. Intracellular Glutathione Quantification

Changes in reduced and oxidized glutathione (GSH) levels in NHBE cells in response to PCN and CoQ9 treatment were measured using the Glutathione Assay Kit (#703002, Cayman Chemical, Ann Arbor, MI, USA) following the manufacturer’s instruction.

### 2.19. Statistical Analysis

Quantitative data were expressed as the mean ± SEM. Student’s *t*-test was used for the comparison between control and each experimental group. Statistical significance was expressed as ns (non-significant), * *p* ≤ 0.05, ** *p* ≤ 0.01, *** *p* ≤ 0.001, or **** *p* ≤ 0.0001.

## 3. Results

### 3.1. CoQ9 from O. raphanipes Protects NHBE Cells Against Pyocyanin-Mediated Cell Death

To examine whether CoQ9 is cytotoxic, NHBE cells were incubated with increasing concentrations of CoQ9 for 24 and 48 h. CoQ9 ranging from 1 to 150 μM did not cause cytotoxicity in NHBE cells (Figure 2A). Also, CoQ9 protects against the PCN-mediated cytotoxicity. The growth of NHBE cells was significantly reduced by 5 and 10 μg/mL PCN at 24 h, but the CoQ9 pretreatment gradually resolved the PCN-induced growth defect in a concentration-dependent manner (Figure 2B).

### 3.2. CoQ9 Restores FOXA2 Expression to Attenuate Mucin Expression in Submerged Pyocyanin-Exposed NHBE Cells

Our previous studies have demonstrated that PCN causes airway mucus hypersecretion by inducing FOXA2 depletion in vivo and in vitro [19,20,22,42]. Based on the previous findings, we examined whether CoQ9 could attenuate PCN-induced mucin overproduction by restoring FOXA2 expression levels in human bronchial epithelial cells. Under submerged culture conditions, CoQ9 gradually restored nuclear FOXA2 levels in a dose-dependent manner in PCN-exposed NHBE cells, with 100 µM CoQ9 maintaining FOXA2 expression comparable to the control group (Figure 3A and Figure S1). Consistent with the restored FOXA2 abundance, 100 µM CoQ9 pretreatment significantly reduced PCN-induced *MUC5AC* and *MUC5B* gene expression by 1.7 and 2.9 fold, respectively (Figure 3B). NHBE cells treated with 100 µM CoQ9 alone did not induce the expression of either *MUC5AC* or *MUC5B* (Figure 3C). By fluorescence-based imaging, we found that PCN increased the expression of MUC5AC and MUC5B mucins by 2.8 and 3.8-fold, respectively (Figure 3D,E). However, 100 µM CoQ9 pretreatment reduced the PCN-induced MUC5AC and MUC5B mucin expression to 1.4 and 1.8-folds relative to the control group, respectively (Figure 3D,E). Consistently, 100 µM CoQ9 pretreatment rescued nuclear FOXA2 expression from PCN-induced depletion to levels similar to the control group (Figure 3A,D,E).

### 3.3. CoQ9 Restores FOXA2 Expression to Attenuate Mucin Expression in Pseudostratified Air–Liquid Interface Cultures of Pyocyanin-Exposed NHBE Cells

We further examined whether CoQ9 mitigates PCN-induced mucin overproduction and FOXA2 depletion in pseudostratified bronchial epithelial cells in ALI culture, recapitulating human bronchial epithelium [73]. By confocal immunofluorescence imaging, we found that PCN increased the expression of MUC5AC and MUC5B in ALI-cultured NHBE cells by 3.6 and 4.4-folds, respectively, whereas CoQ9 pretreatment suppressed the expression of MUC5AC and MUC5B to 1.7 and 1.8-fold, respectively, relative to the control groups (Figure 4A,B, value set to 1x). Conversely, PCN decreased FOXA2 abundance to 0.47 and 0.61 folds, respectively. However, CoQ9 pretreatment restored FOXA2 expression levels under PCN exposure to the control group levels (Figure 4A,B). We further examined whether CoQ9 modulates the mucin gene expression transcriptionally. PCN exposure elevated the mRNA level of *MUC5AC* and *MUC5B* by 2.5 and 2.2 folds, respectively, whereas CoQ9 pretreatment downregulated the expression of *MUC5AC* and *MUC5B* t0 1.78 and 1.61-folds, respectively, relative to the control groups (Figure 4C). In contrast, *FOXA2* expression was depleted by PCN by 0.21-fold but restored to the basal expression levels of the control group by CoQ9 pretreatment (Figure 4C). Collectively, our data suggest that CoQ9 attenuates PCN-induced goblet cell hyperplasia and mucin hyperproduction by restoring FOXA2 expression and function.

### 3.4. CoQ9 Downregulates Pyocyanin-Induced EGFR and STAT6 Signaling Pathways

Because CoQ9 mitigated mucin biosynthesis and restored FOXA2 expression in PCN-exposed NHBE cells, we examined whether CoQ9 modulates PCN-induced mucin-promoting signaling pathways, the EGFR-AKT/ERK1/2 and STAT6-SPDEF axes, which directly repress FOXA2 activation and transcriptional expression, respectively [19,20,22,25]. PCN increased the activation of pEGFR-pAKT/pERK1/2, which was blocked by CoQ9 pretreatment (Figure 5A and Figure S2). Similarly, nuclear pSTAT6 and nuclear SPDEF protein abundance were increased by PCN but attenuated by CoQ9 pretreatment (Figure 5B and Figure S3). Intriguingly, NHBE cells treated with CoQ9 alone in the absence of PCN increased the level of pEGFR, and pAKT (Figure 5A and Figure S2), suggesting that CoQ9, similar to CoQ10, impacts other cellular processes such as cell migration and wound healing through activating PI3K/AKT-mTOR and ERK1/2 [74,75]. Collectively, the aforementioned data demonstrate that CoQ9 extensively suppresses PCN-induced mucin-producing signaling pathways.

### 3.5. CoQ9 Reduces Pyocyanin-Imposed Mitochondrial Oxidative Stress by Enhancing the SIRT3-SOD2 Axis

As we have discussed above, PCN-induced oxidative stress activates mucin-producing signaling cascades (Figure 5) [19,36,40,41,43]. We hypothesized that CoQ9 may attenuate oxidative stress in PCN-exposed NHBE cells. To examine whether CoQ9 has antioxidant properties, we conducted a DPPH assay in comparison to ascorbic acid, a potent reducing agent. CoQ9 scavenged DPPH radicals in a dose-dependent manner, with 100 μM CoQ9 exhibiting an approximately 30% free radical scavenging effect compared to the same amount of ascorbic acid (Figure 6A). Additionally, CoQ9 pretreatment significantly mitigated intracellular ROS generation in PCN-exposed NHBE cells (Figure 6B).

Because PCN activates redox cycling by hijacking electrons from reduced glutathione (GSH) [45], we next investigated whether CoQ9 modulates GSH-mediated antioxidant defense. We examined the intracellular levels of reduced and oxidized glutathione (GSH and GSSG). PCN exposure significantly decreased total glutathione levels (GSH+GSSG) (Figure 6C, upper panel). Although CoQ9 pretreatment induced an increasing trend in total glutathione levels in PCN -exposed NHBE cells, it was not statistically significant compared to the basal levels in the control group (Figure 6C, upper panel). Conversely, PCN significantly increased GSSG levels in NHBE cells, which were suppressed by CoQ9 pretreatment (Figure 6C, bottom panel), suggesting that GSH levels in the total glutathione pool are relatively higher in CoQ9-pretreated cells, compared to the PCN-exposed group. Next, we examined whether CoQ9 pretreatment enhanced glutathione de novo biosynthesis through the increase in γ-glutamylcysteine synthetase (γGCS) [76], a rate-limiting enzyme for glutathione synthesis. A component of the γGCS holoenzyme, glutamate-cysteine ligase catalytic subunit (*GCLS*) mRNA expression was significantly induced by PCN, likely indicating the cells were trying to detoxify PCN-generated ROS (Figure 6D). Significantly, provision of CoQ9 reduced the *GCLS* expression during PCN exposure, although not at a statistically significant level (Figure 6D), suggesting that CoQ9, together with glutathione-dependent antioxidant defense, synergistically attenuates PCN-induced cellular damage. In addition, heme oxygenase-1 (*HO-1*) mRNA expression was simultaneously mitigated by CoQ9 pretreatment without statistical significance (Figure 6D). These findings imply that CoQ9 may regulate the antioxidant defense system at transcription levels.

Next, we examined how CoQ9 could modulate the expression of NRF2, the master transcriptional regulator of cellular redox homeostasis. We showed that CoQ9 increased the nuclear abundance of NRF2 after PCN exposure (Figure 6E and Figure S4), suggesting that CoQ9 could protect antioxidant enzymes from destabilization by PCN-mediated redox cycling. To test this possibility, we examined the expression of glutathione peroxidase (GPX4), a strictly GSH-dependent antioxidant enzyme that prevents lipid peroxidation and ferroptosis [77], which is depleted in PCN-exposed NCI-H292 mucoepidermoid cells [56]. Consistent with previous studies, GPX4 protein expression was significantly downregulated by PCN, whereas CoQ9 pretreatment preserved GPX4 expression (Figure 6E and Figure S4). These findings suggest that CoQ9 rescues PCN-exposed NHBE cells by its own intrinsic antioxidant properties, as well as supporting GSH homeostasis.

### 3.6. CoQ9 Maintains Mitochondrial Fidelity and Biogenesis Under PCN-Exerted Oxidative Stress

Exogenous provision of CoQ9 robustly reduced GSSG levels while it moderately restored total glutathione levels (Figure 6C), suggesting that CoQ9 raises cellular antioxidant potential by preserving the GSH pool rather than by promoting GSH biosynthesis. Although GSH recycling largely occurs in the cytosol, mitochondrial GSH recycling is crucial for preventing mitochondrial dysfunction from oxidative stress, e.g., by PCN [48]. We investigated whether CoQ9 could protect the mitochondrial antioxidant defense system from PCN-induced superoxide generation. PCN increased superoxide generation by 2.6-fold compared to the untreated control, whereas CoQ9 pretreatment abrogated superoxide generation in PCN-exposed NHBE cells (Figure 7A). Consistent with mitochondrial ROS levels, CoQ9 treatment restored mitochondrial membrane potential disrupted by PCN (Figure 7B). Compared to the control group, PCN-exposed NHBE cells significantly increased JC-1 monomer levels (green), while decreasing JC-1 dimer (Red). In contrast, CoQ9 treatment mitigated JC-1 monomer conversion from the dimer, indicating that CoQ9 protects mitochondria from PCN-induced membrane leakage. Since mitochondrial dysfunction induces a metabolic shift toward glycolysis, we further assessed lactate production levels, a byproduct of glycolysis. Interestingly, despite reduced mitochondrial membrane potential, lactate production was significantly decreased by PCN alone, whereas CoQ9 pretreatment restored lactate levels similar to the control group (Figure 7C). Consistent with lactate production levels, PCN-mediated mitochondrial dysfunction was further confirmed by ATP deprivation in NHBE cells, which was reversed by CoQ9 (Figure 7D), suggesting that CoQ9 improves mitochondrial redox balance.

Mitochondrial redox regulation is centered on Sirtuin 3 (SIRT3)-mediated deacetylation of antioxidant and metabolic enzymes [52]. Because PCN-induced mitochondrial superoxide levels were significantly downregulated by CoQ9, we examined whether the expression and activation of superoxide dismutase 2 (SOD2), an initial mitochondria-specific antioxidant enzyme that converts superoxide to hydrogen peroxide, were modulated by CoQ9. PCN exposure triggered *SOD2* gene expression, which was significantly reduced by CoQ9 (Figure 7E). In contrast, *SIRT3* was stably expressed with insignificant changes (Figure 7E). Interestingly, PCN significantly decreased the SOD2 protein expression, whereas CoQ9 blocked this depletion (Figure 7F and Figure S5). Because SOD2 activity is tightly regulated by SIRT3-mediated deacetylation [54,55], we evaluated the correlation between SOD2 acetylation levels and SIRT3 expression. PCN exposure significantly lowered SIRT3 protein abundance with concomitant increase in SOD2 acetylation. Importantly, CoQ9 restored the expression of SIRT3 and SOD2 depleted by PCN, with concomitant decrease in SOD2 acetylation levels (Figure 7F and Figure S5), supporting that CoQ9 mitigates PCN-mediated mitochondrial dysfunction by preserving SIRT3-SOD2 mitochondrial antioxidant barrier.

Mitochondrial remodeling is important for functional recovery, such as improving energy production efficiency. Mitophagy is a cellular process that improves mitochondrial function by eliminating damaged mitochondria through autophagy [78]. PINK1 is a specific biomarker for mitophagy induction, which mediates autophagosome formation in damaged mitochondria [78]. We found that PINK1 abundance was substantially diminished by PCN, which was mitigated by CoQ9 (Figure 7G and Figure S6). In contrast to PINK1, Parkin, an E3 ubiquitin-protein ligase that tags damaged mitochondria for mitophagy, was consistently expressed independent of CoQ9 and PCN (Figure 7G). In comparison with TOM20, a marker for mitochondrial mass, relative expression of both PINK1 and Parkin was decreased (Figure 7G and Figure S6). LC3B is an essential component for the formation of an autophagosome by targeting the tagging proteins, PINK1 and Parkin, on the outer mitochondrial membrane [79]. In PCN-exposed NHBE cells, both LC3B-I (top band) and LC-3B-II (bottom band) were increased (Figure 7G and Figure S6). The cells treated with CoQ9 alone induced both LC3B-I and LC3B-II, which were additionally enhanced by PCN exposure (Figure 7G and Figure S6). These findings suggest that CoQ9 restores mitochondrial integrity by improving defective mitophagy and improving mitochondrial function under PCN exposure.

Figure 7D shows CoQ9 pretreatment replenishes ATP levels in PCN-exposed NHBE cells, suggesting that, in addition to restoring the SIRT3-SOD2 antioxidant system and mitophagy process, CoQ9 also helps in maintaining functional mitochondria. Because the cells compensate for mitophagy-induced energy deficiency by activating the mitochondrial biogenesis [80], we further examined whether CoQ9 enhances the AMPK-PGC1α axis, a core signaling cascade for mitochondrial biogenesis. PCN exposure activates pPGC1α and pAMPK but downregulates their total protein abundance (Figure 7H and Figure S7). The upregulation of pPGC1α and pAMPK by PCN likely reflects the attempt by NHBE cells to enhance mitochondrial biogenesis to replenish damaged mitochondria. Interestingly, CoQ9 pretreatment alone also activates pPGC1α and pAMPK independent of PCN, and more importantly, CoQ9 restored the protein abundance of PGC1α and AMPK under PCN exposure (Figure 7H and Figure S7). Given the regulatory effects of CoQ9 on mitochondrial redox balancing, mitophagy, and mitochondrial biogenesis in the presence of PCN, our data suggest that CoQ9 minimized pathophysiological redox cycling of PCN in mucin overproduction and goblet cell hyperplasia by protecting mitochondrial fidelity from severe oxidative stress in human bronchial epithelial cells.

## 4. Discussion

PCN secreted from *P. aeruginosa* induces goblet cell hyperplasia and mucus hypersecretion by inducing chronic oxidative stress in airway epithelial cells [19,25,41,42]. The redox cycling properties of pyocyanin overwhelm the capacity of antioxidants and antioxidative enzymes of the host cells. The redox imbalance disrupts normal mitochondrial function, a critical step in establishing the vicious cycle between chronic oxidative stress and mucin hyperproduction [25,38,47,48,56]. In this study, we demonstrate that CoQ9 purified from *O. raphanipes* mitigates PCN-induced goblet cell hyperplasia and mucin overproduction by reducing oxidative stress and by restoring mitochondrial fidelity.

As a homolog of CoQ10, CoQ9 is an essential ubiquinone, mainly acting as an electron carrier and redox mediator in the mitochondrial oxidative phosphorylation machinery [81]. In addition to ATP generation, CoQ9 maintains redox homeostasis with its ROS scavenging properties, which resemble the physiological roles of CoQ10 [68,69]. The intracellular ratio between CoQ9 and CoQ10 varies across different species. Accordingly, the dominant coenzyme homolog is primarily utilized over the other ubiquinones in neutralizing ROS. In mammals, CoQ9 is abundant in mouse and rat, whereas CoQ10 is the predominant in human, guinea pig, and rabbit [82]. Under 2,2′-azobis (2-amidinopropane) dihydrochloride (AAPH)-induced oxidative stress, rat hepatocytes with total CoQ9 and CoQ10 at a ratio of 6:1 primarily spend the reduced CoQ9 for antioxidative process [83]. In contrast, guinea pig hepatocytes with total CoQ9 and CoQ10 at a ratio of 1:5 show a substantial decrease in reduced CoQ10 (ubiquinol) levels. Interestingly, CoQ9 levels decrease concurrently with the depletion of ubiquinol and the accumulation of oxidized CoQ10 (ubiquinone) [83], implying that CoQ9 actively supports the physiological functions and redox cycling of CoQ10. Consequently, in organisms that are predominantly dependent on CoQ10, exogenous CoQ9 supplementation compensates for the CoQ10 deficits and sustains ubiquinone homeostasis via direct metabolic conversion during the period of severe cellular stress. In ischemic reperfusion guinea pig models, CoQ9 supplementation protects hearts from ischemia–reperfusion by mitigating cardiomyocyte death, to a level comparable to CoQ10 [65]. These findings indicate that CoQ10-dependent organisms can utilize exogenous CoQ9, thereby compensating for a loss of CoQ10 under cellular stress.

We have previously shown that PCN triggers mucin overproduction and goblet cell hyperplasia by depleting FOXA2, a key transcriptional repressor of *MUC5AC* and *MUC5B*, via the activation of the EGFR-AKT/ERK1/2 and STAT6-SPDEF axes [19,20,22,25]. PCN-induced oxidative stress causes the oxidation-driven hyperactivation of EGFR and STAT6 [19,84,85,86], that converge to inactivate FOXA2 [19,26,27]. Because intracellular antioxidants are rapidly overwhelmed by the redox-cycling properties of PCN, antioxidant supplementation inactivates EGFR and STAT6 and restores FOXA2 expression to attenuate mucin production [19,41]. This suggests that neutralizing ROS via exogenous antioxidant supplementation such as GSH and *N*-acetyl cysteine (NAC) is a promising mucoregulatory therapeutic option to alleviate mucus hypersecretion under chronic oxidative stress conditions. Consistent with this, previous [64,65,68,69,83] and current studies have demonstrated the protective effects of CoQ9 against oxidative stress-induced injury due to its antioxidant properties, indicating that the suppressive effects of CoQ9 on PCN-induced mucin overproduction are principally mediated by the reduction in oxidative stress.

Previous studies have shown PCN oxidizes NADPH and GSH, two major metabolites that comprise the mitochondrial antioxidant recycling system [45,87], leading to mitochondrial dysfunction. Under normoxia or mild oxidative stress, NADPH acts as an electron donor to reduce GSSG to GSH within the mitochondria [88]. However, in the presence of PCN, both metabolites are primarily spent by the redox cycling of PCN, which impairs the reduction in GSSG and NADP+ [38,45,89]. This loss of NADPH and GSH exacerbates intracellular oxidative stress via superoxide leakage from damaged mitochondria, accompanied by increased mitochondrial membrane permeability [90]. Notably, increased mitochondrial membrane permeability is positively correlated with the induction of mucin biosynthesis. In NCI-H292 human mucoepidermoid pulmonary cells, hydrogen peroxide-induced oxidative stress increases mitochondrial permeability and triggers the release of mitochondrial DNA (mtDNA) into the cytoplasm. This cytosolic mtDNA activates the cGAS-STING pathway, which senses misplaced DNA, thereby driving mucin overproduction in coordination with EGFR activation [91]. Consistent with this line of thought, the current study demonstrates that CoQ9 pretreatment reduces mitochondrial superoxide generation and restores ATP production in PCN-exposed NHBE cells. Taken together, these findings indicate that the mucoregulatory function of CoQ9 is mediated by the preservation of mitochondrial integrity and fidelity against PCN-induced oxidative stress.

Mitochondrial integrity and fidelity are tightly regulated by SIRT3, a mitochondria-specific NAD^+^-dependent histone deacetylase [52]. SIRT3 simultaneously regulates the activation of rate-limiting enzymes in various metabolic pathways and maintains mitochondrial antioxidant capacity by regulating antioxidant recycling and production, as well as antioxidative enzyme function and stability. For instance, during the NADPH-dependent GSSG-GSH recycling, SIRT3-mediated deacetylation of isocitrate dehydrogenase 2 (IDH2) upregulates NADPH biosynthesis, thereby increasing reduced GSH levels [88]. Additionally, SIRT3-mediated deacetylation at lysine 68 and 122 of SOD2 is a critical step for enzymatic activation and the subsequent conversion of superoxide into hydrogen peroxide [54]. Consistently, overexpression of *SIRT3* or deacetylation-mimicking *SOD2* mutant (*SOD2*-K53/89R) extensively reduces ROS generation [54], whereas a lack of SIRT3 expression elevates mitochondrial oxidative stress due to reduced SOD2 activities [53]. In the context of PCN-induced oxidative stress and mucin overproduction, SIRT3 activation by a specific agonist, ganoderic acid D, hinders airway mucin production via activation of the NRF2-GPX4 axis [56]. This suggests that the SIRT3-SOD2 mitochondrial antioxidant machinery limits mucin overproduction in the presence of PCN. Notably, CoQ9 restores SIRT3 and SOD2 expression depleted by PCN exposure, suggesting that CoQ9 suppresses PCN-induced mucin overproduction by detoxifying mitochondrial superoxide via the restoration of SIRT3-SOD2 signaling. Intriguingly, mRNA and protein expression levels of SIRT3 and SOD2 were poorly correlated in PCN and CoQ9-treated NHBE cells. Although NRF2 is a key transcription factor regulating both *SIRT3* and *SOD2* gene expression, nuclear NRF2 protein levels did not correlate with *SIRT3* expression. In contrast, *SOD2* mRNA levels trended similarly to nuclear NRF2 proteins. However, SOD2 mRNA and protein showed opposite expression patterns under PCN exposure. This discrepancy between mRNA and protein levels strongly implies the involvement of post-translational modification (PTMs). Previous studies have shown that SIRT3 and SOD2 proteins can be destabilized by PTMs under chronic or severe oxidative stress [92,93]. Our findings suggest that CoQ9 pretreatment prevents PCN-induced destabilization of SIRT3 and SOD2 proteins by mitigating mitochondrial oxidative stress. Alongside PTMs, posttranscriptional regulation caused by ER stress and stress granule formation under oxidative conditions may contribute to these distinct protein profiles. The precise mechanisms governing SIRT3 and SOD2 regulation at the post-transcription and post-translation levels under PCN-induced oxidative stress remain to be elucidated in future studies.

In parallel with restoring the mitochondrial antioxidant system, our data show that CoQ9 abrogated PCN-mediated PINK1 depletion. PINK1 initiates mitophagy by gathering on the outer membrane of mitochondria and subsequently recruiting PARKIN, a ubiquitin ligase that tags damaged mitochondria for clearance [94]. As *PINK1* expression is regulated by NRF2 [95] and FOXO3a [96], PINK1 expression is increased in response to oxidative stress, enabling the rapid disassembly of damaged mitochondria through facilitating mitophagy. Interestingly, we found that PINK1 abundance was substantially decreased in PCN-exposed NHBE cells, which may be caused by ATP deprivation and a loss of SIRT3. Previous studies have demonstrated that ATP shortage strongly downregulates PINK1 expression by impairing *PINK1* mRNA translation independent of changes in gene expression and posttranslational modification [97]. Additionally, *SIRT3* knockdown decreases PINK1 expression and stability [98], suggesting that concurrent loss of both ATP and SIRT3 in PCN-exposed NHBE cells synergistically promotes PINK1 depletion. This loss of PINK1 subsequently downregulates mitochondrial membrane potential and accelerates superoxide leakage from damaged mitochondria, ultimately leading to mitochondrial dysfunction through the inactivation of the mitochondrial respiratory chain [99]. This chain reaction allows PCN to establish persistent intracellular oxidative stress by creating a vicious cycle between mitochondrial damage and continuous ROS generation. In turn, this sustained ROS accumulation activates the EGFR and STAT6 signaling cascades that inhibit FOXA2, ultimately causing mucin overproduction and goblet cell hyperplasia. In PCN-exposed NHBE cells, CoQ9 pretreatment preserved PINK1 expression while increasing mitochondrial membrane potential and the expression of SIRT3, nuclear NRF2, and ATP levels. Furthermore, the expression of LC3B-I and LC3B-II was significantly elevated. Collectively, these findings indicate that CoQ9 mitigates PCN-induced mitochondrial superoxide damage by enhancing the mitochondrial quality control system.

Apart from restoring the mitochondrial quality control system, CoQ9 enhances mitochondrial biogenesis by activating the AMPK-PGC1α axis, a canonical signaling cascade triggered in response to energy demand [80]. Intriguingly, both PCN and CoQ9 activate this pathway, but via distinct mechanisms. Under PCN exposure, airway epithelial cells undergo ATP deprivation [48], a potent trigger for AMPK activation. However, despite initial mitochondrial biogenesis, the persistent oxidative stress caused by redox cycling of PCN, coupled with the accumulation of damaged mitochondria, compromises energy production efficiency, culminating in a chronic energy deficit. In contrast, CoQ9 activates mitochondrial biogenesis either through direct AMPK activation or by elevating endogenous CoQ10 levels. As a homolog of CoQ10, CoQ9 shares structural and functional similarities that allow it to act as an upstream activator of the AMPK-PGC1α signaling. Consistent with the documented agonistic effects of CoQ10 on the AMPK-PGC1α signaling [100,101,102], CoQ9 induces the phosphorylation of AMPK at Thr172 and PGC1α at Ser571 in NHBE cells. Furthermore, exogenous CoQ9 supplementation elevates CoQ10 levels via direct conversion as observed in guinea pigs [64,65], suggesting that CoQ9 supplementation could effectively elevate CoQ10 levels in humans, who share a similar CoQ10 dependency. Under PCN-induced oxidative stress, CoQ9-pretreated NHBE cells sustained AMPK-PGC1α activation at levels comparable to the CoQ9 alone-treatment group. Reflected by the restored ratio of PINK1 to TOM20 alongside restored ATP levels, these data demonstrate that exogenously provided *O. raphanipes* CoQ9 contributes to the maintenance of a functional mitochondrial pool via coordinated mitophagy and biogenesis. This process is critical for sustaining the intracellular antioxidant reservoir, thereby protecting NHBE cells against PCN-induced oxidative stress and uncontrolled mucus hypersecretion.

## 5. Conclusions

CoQ9 derived from *O. raphanipes* protects bronchial epithelial cells from PCN-induced oxidative stress by preserving mitochondrial integrity and fidelity. PCN-induced mitochondrial superoxide damage causes airway goblet cell hyperplasia by causing persistent oxidative stress and creating vicious cycles between damaging mitochondria and continuous ROS generation, gradually shrinking host antioxidant capacities and ATP pool due to defective mitochondrial quality control systems. CoQ9 preserves ATP and antioxidant reservoirs from redox cycling of PCN by protecting mitochondrial redox homeostasis through stabilizing SIRT3-SOD2 antioxidant defense, which further improves mitochondrial quality in conjunction with mitophagy and mitochondrial biogenesis (Figure 8). Our data suggest an alternative approach to improve the detoxifying efficiency of antioxidant defense redox in airway epithelial cells damaged by oxidative stress with ubiquinone homolog.

## Figures and Tables

**Figure 1:**
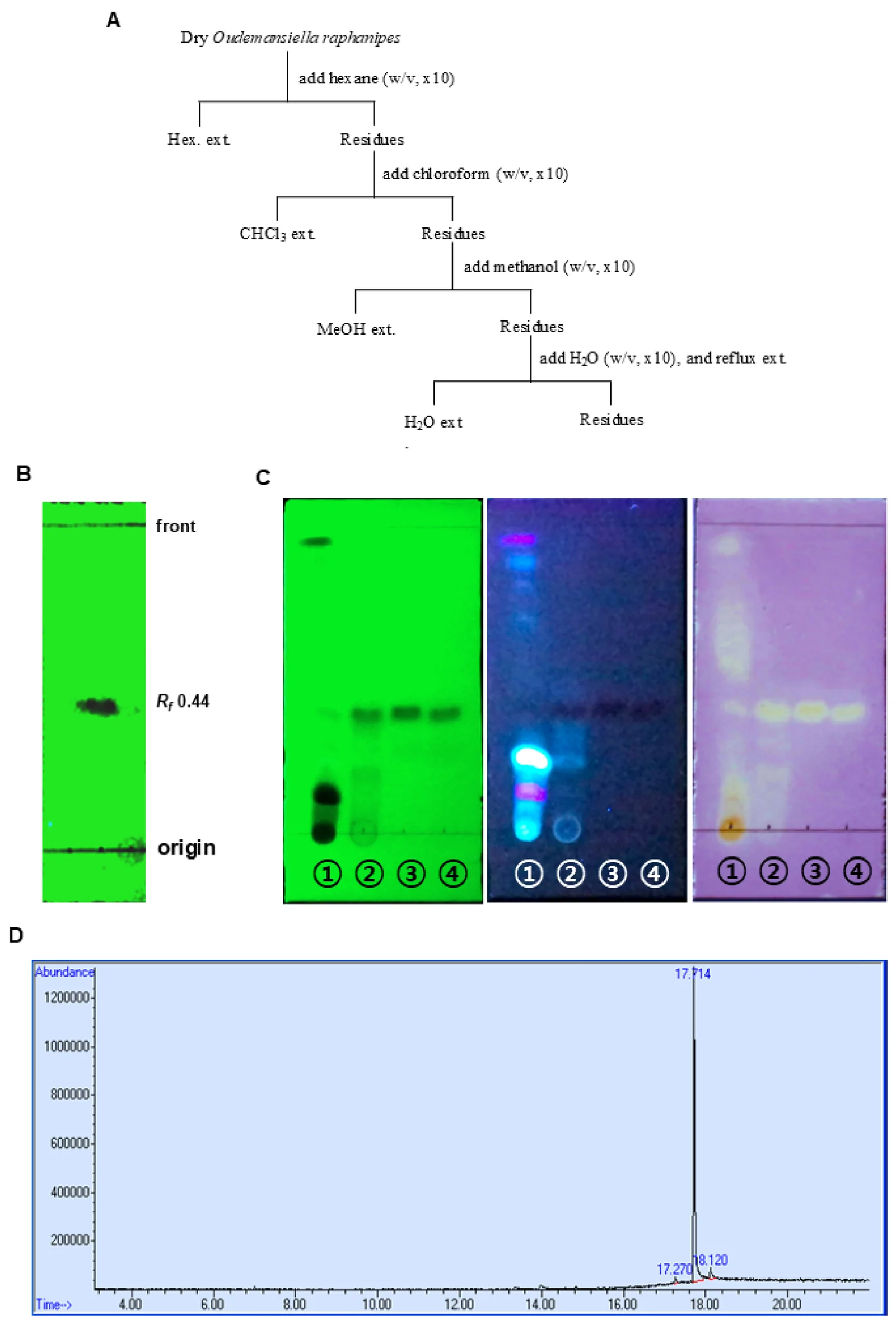
CoQ9 purification from *O. raphanipes*. (**A**) Schematic diagram of stepwise extraction of extracts. (**B**,**C**) TLC developing patterns. (**B**) TLC chromatogram of purified compound (R*_f_* 0.44, Developing solvent; hexane:ethylacetate (10:1)). (**C**) TLC chromatography for CoQ9 from *O. raphanipes.* CoQ9 contents in fractionized extracts from chromatography were visualized at UV 254 nm, 366 nm and 520 nm. Each lane indicates the following molecules: ① 1st column chromatography fractionized compounds from *O. raphanipes*, ② 1st TLC band 2 compound, ③ CoQ-9 purified from *O. raphanipes* extracts and ④ standard CoQ-9. (**D**) FID-GC chromatogram of the purified compound (R*t* 17.714).

**Figure 2:**
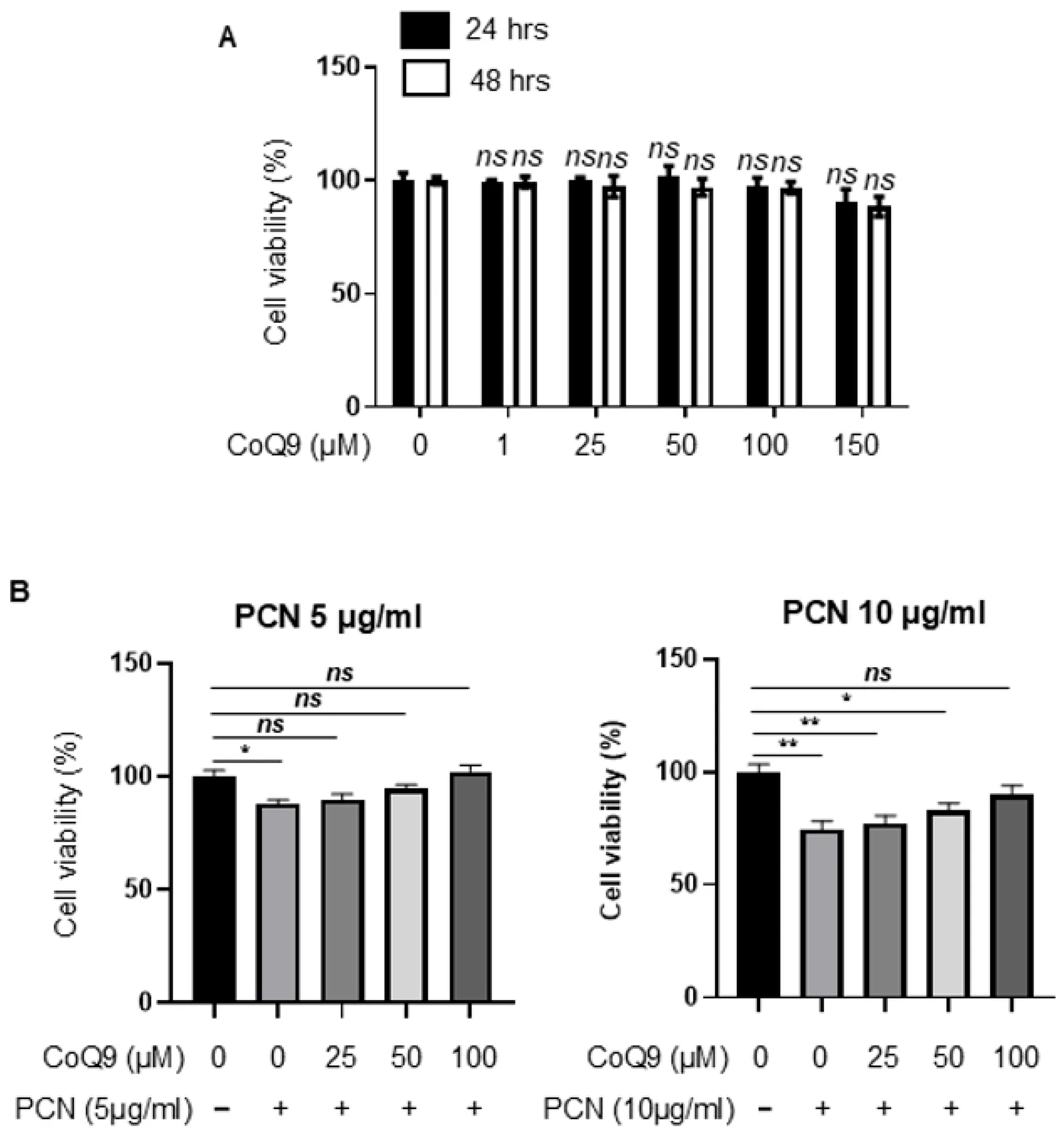
Viability of NHBE cells in the presence of CoQ9 and pyocyanin. NHBE cells were cultured in 96-well plates for 24 h and pretreated with CoQ9 for 1 h before exposure to pyocyanin for 24 or 48 h. After the treatment, the cells were incubated with WST-1 reagent to measure the cell viability. (**A**) Viability of NHBE cells under incubation with CoQ9 (1, 25, 50, and 100 μM) for 24 and 48 h. (**B**) Viability of NHBE cells in the presence of pyocyanin (5 and 10 μg/mL) and CoQ9 (25, 50, and 100 μM) for 24 h. Data are presented as ± SEM (n = 3). * *p* < 0.05, ** *p* < 0.01, *ns* = not significant by Student’s *t*-test.

**Figure 3:**
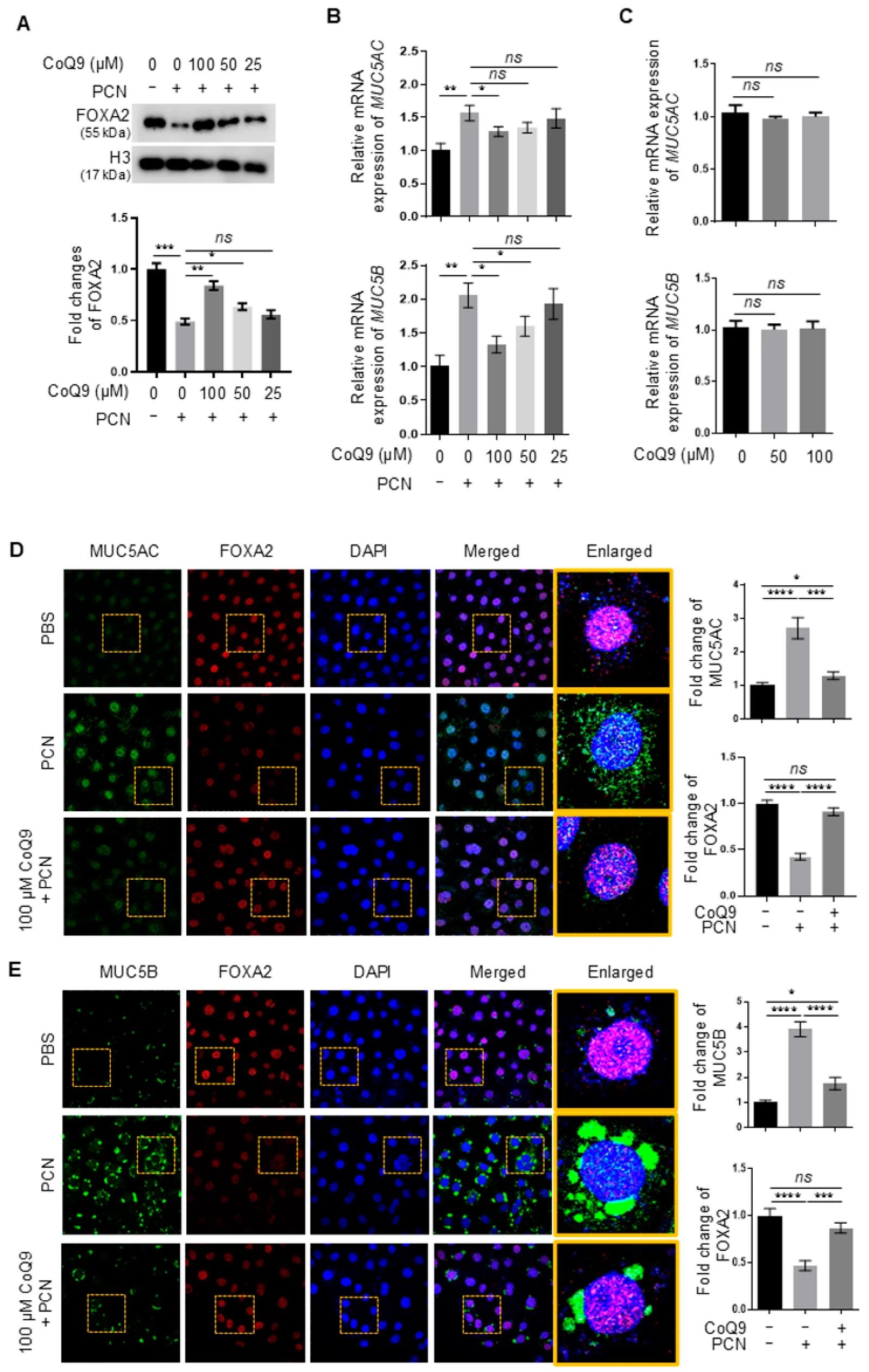
Changes in expression of mucins and FOXA2 within PCN- and CoQ9-treated NHBE cells under submerged culture conditions. NHBE cells were preincubated with the indicated concentrations of CoQ9 for 1 h and subsequently exposed to 5 μg/mL pyocyanin for 24 h. (**A**) Western blot analysis of nuclear FOXA2 expression normalized to histone H3, and the relative FOXA2 expression was plotted. (**B**) Relative mRNA expression of *MUC5AC* and *MUC5B* from qPCR. (**C**) *MUC5AC* and *MUC5B* expression in NHBE cells treated with CoQ9 alone in a dose-dependent manner. (**D**,**E**) Immunofluorescence labeling of MUC5AC, MUC5B, and FOXA2. MUC5AC and MUC5B were visualized using Alexa Fluor 488 (Green), and FOXA2 was visualized by Alexa Fluor 594 (Red). Nuclei were counterstained with DAPI. The region of interest (yellow dashed boxes) are shown as enlarged images in the right panels. The fluorescent intensities of MUC5AC, MUC5B, and FOXA2 were quantified and normalized to the control group. Enlarged images display the cells in the box with dashed lines. Data are presented as ±SEM (n = 3). * *p* < 0.05, ** *p* < 0.01, *** *p* < 0.001, **** *p* < 0.0001, *ns =* not significant by Student’s *t*-test.

**Figure 4:**
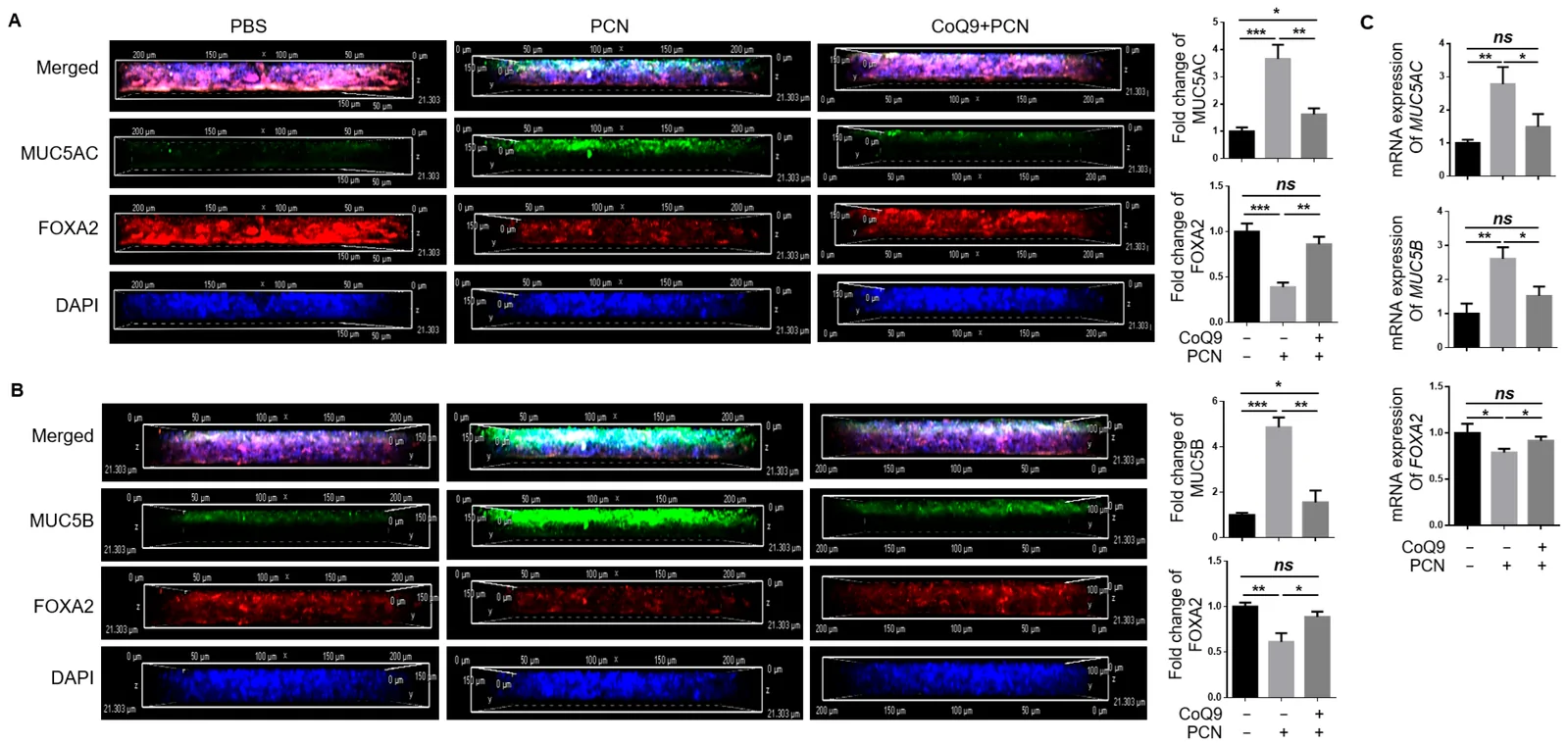
Regulatory effects of CoQ9 on expression of mucins and FOXA2 in ALI-cultured NHBE cells. NHBE cells were allowed to differentiate for 21 days in ALI culture and subsequently preincubated with 100 μM CoQ9 for 1 h prior to exposure to 5 μg/mL pyocyanin for 24 h. (**A**,**B**) Immunofluorescence staining of MUC5AC, MUC5B, and FOXA2 in Z-stack images. MUC5AC and MUC5B were probed using Alexa Fluor 488 (Green), and FOXA2 was probed using Alexa Fluor 594 (Red). Nuclei were counterstained with DAPI. Fluorescence intensities of MUC5AC, MUC5B, and FOXA2 were quantified using ImageJ. (**C**) Relative mRNA levels of *MUC5AC*, *MUC5B*, and *FOXA2* genes were quantified by qPCR. All quantified values from both immunofluorescence staining and qPCR measurements were normalized to the untreated control group. Data are presented as mean ± SEM (n = 3). * *p* < 0.05, ** *p* < 0.01, *** *p* < 0.001, *ns =* not significant by Student’s *t*-test.

**Figure 5:**
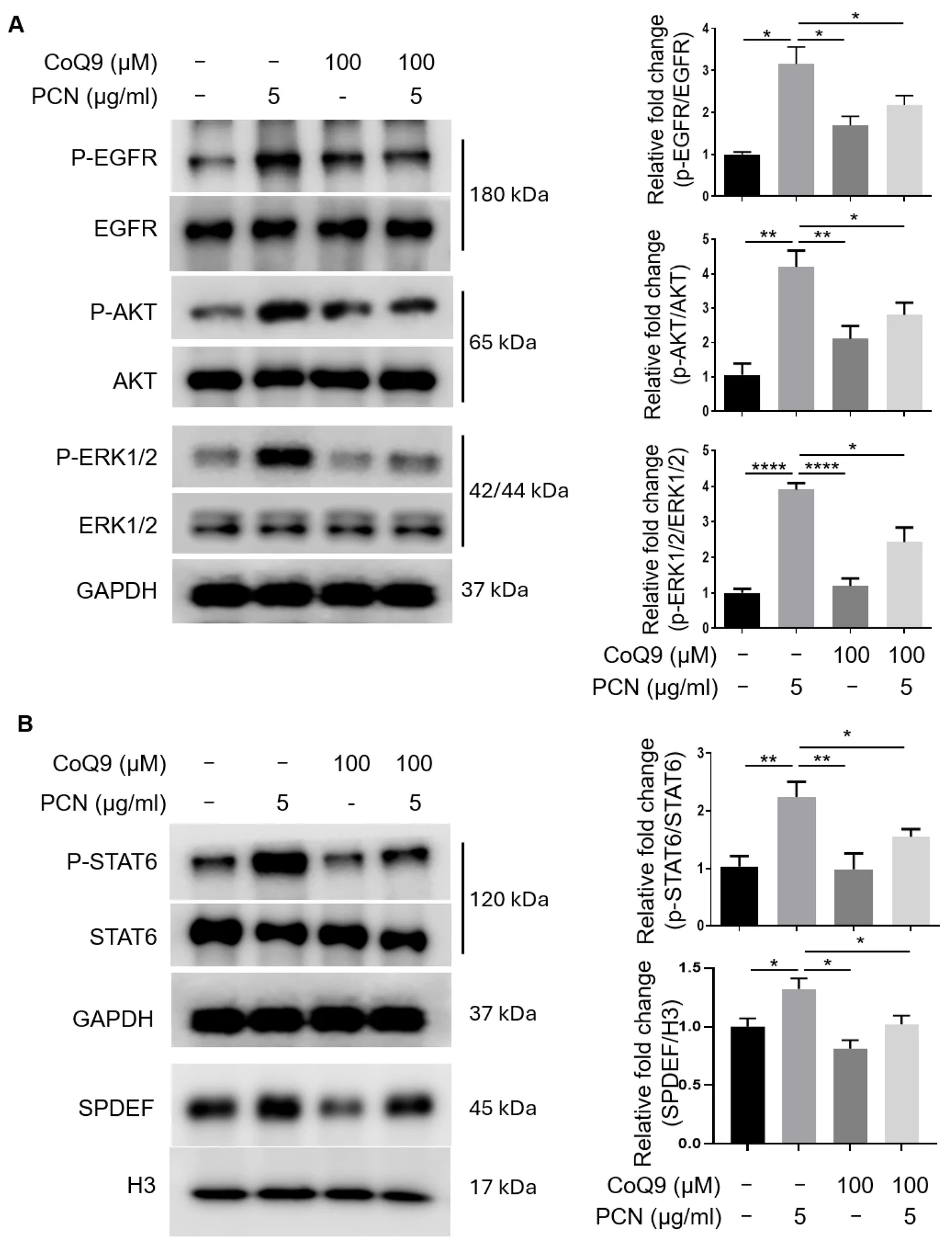
Regulatory effects of CoQ9 on EGFR and STAT6 signaling cascades in NHBE cells exposed to pyocyanin. Submerged NHBE cells were pretreated with 100 µM CoQ9 for 1 h before exposure to 5 µg/mL pyocyanin for 24 h. (**A**) Western blot analysis of total and phosphorylated EGFR, AKT, and ERK1/2 in whole cell lysates. (**B**) Western blot analysis of total and phosphorylated STAT6 in whole cell lysates, and SPDEF levels in nuclear protein extracts. Densitometric analyses of all blots were quantified using ImageJ, and protein expression values were normalized to the untreated control group. Data are presented as mean ± SEM (n = 3). * *p* < 0.05, ** *p* < 0.01, **** *p* < 0.0001, *ns =* not significant by Student’s *t*-test.

**Figure 6:**
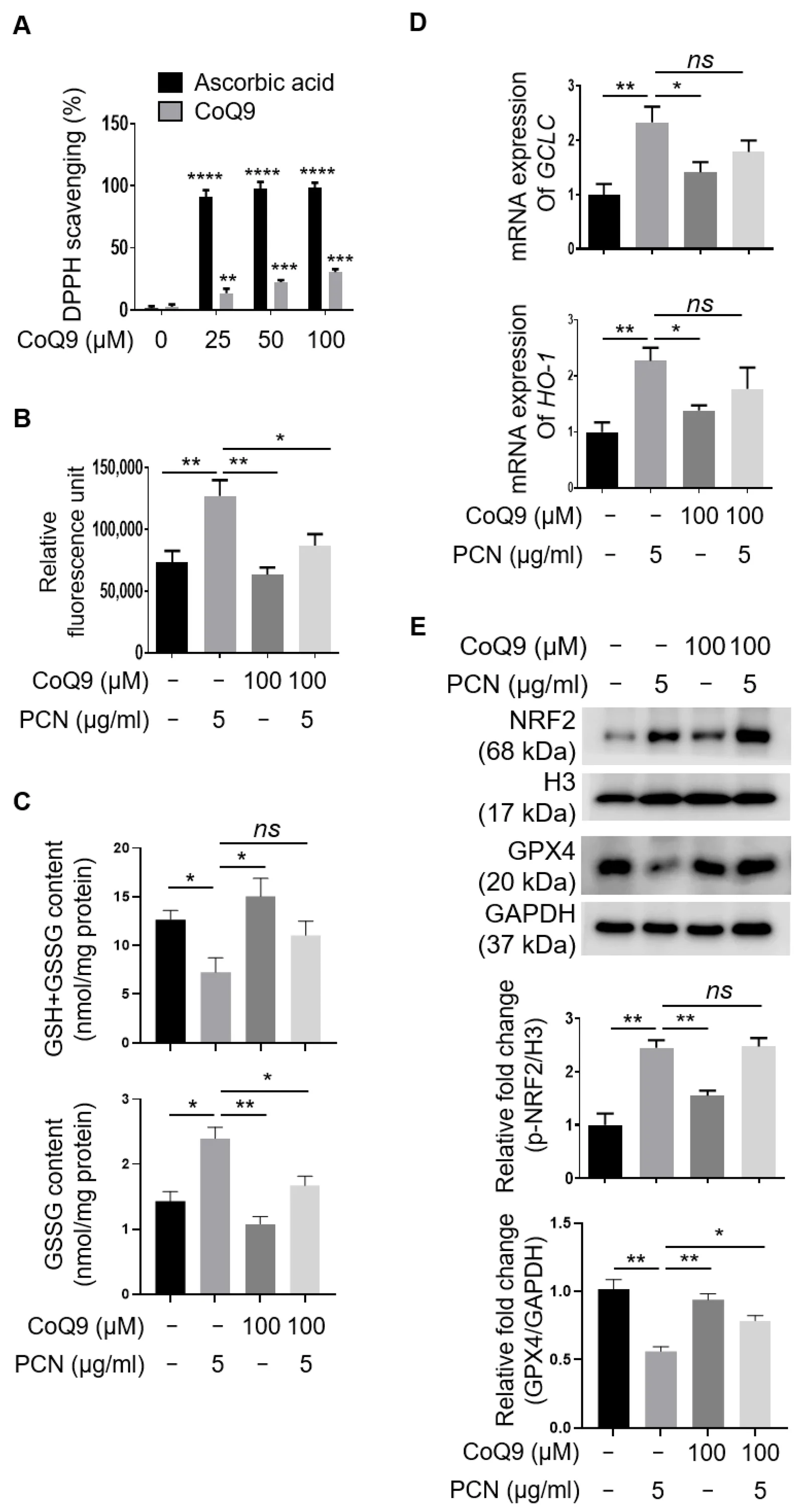
Antioxidant properties of CoQ9 in PCN-exposed NHBE cells. (**A**) DPPH scavenging assay assessing the antioxidant potency of CoQ9 (25, 50, and 100 µM). Ascorbic acid was used at equivalent concentrations as a positive antioxidant control to measure the relative scavenging activity of CoQ9. (**B–E**) NHBE cells cultured under submerged conditions were pretreated with 100 μM CoQ9 for 1 h prior to exposure to 5 μg/mL PCN for 24 h. (**B**) Quantification of intracellular ROS levels. (**C**) Measurement of intracellular total glutathione (GSH+GSSG; top panel) and oxidized glutathione (GSSG; bottom panel) levels. (**D**) mRNA expression of *GCLC* and *HO-1* as measured by qPCR. (**E**) Western blot analysis of nuclear NRF2 and whole-cell GPX4. Histone H3 and GAPDH were used as loading controls for nuclear and whole cell lysates. Immune blots were quantified by ImageJ, and the relative intensities were normalized to the untreated control group. Data are presented as mean ± SEM (n = 3). * *p* < 0.05, ** *p* < 0.01, *** *p* < 0.001, **** *p* < 0.0001, *ns =* not significant by Student’s *t*-test.

**Figure 7:**
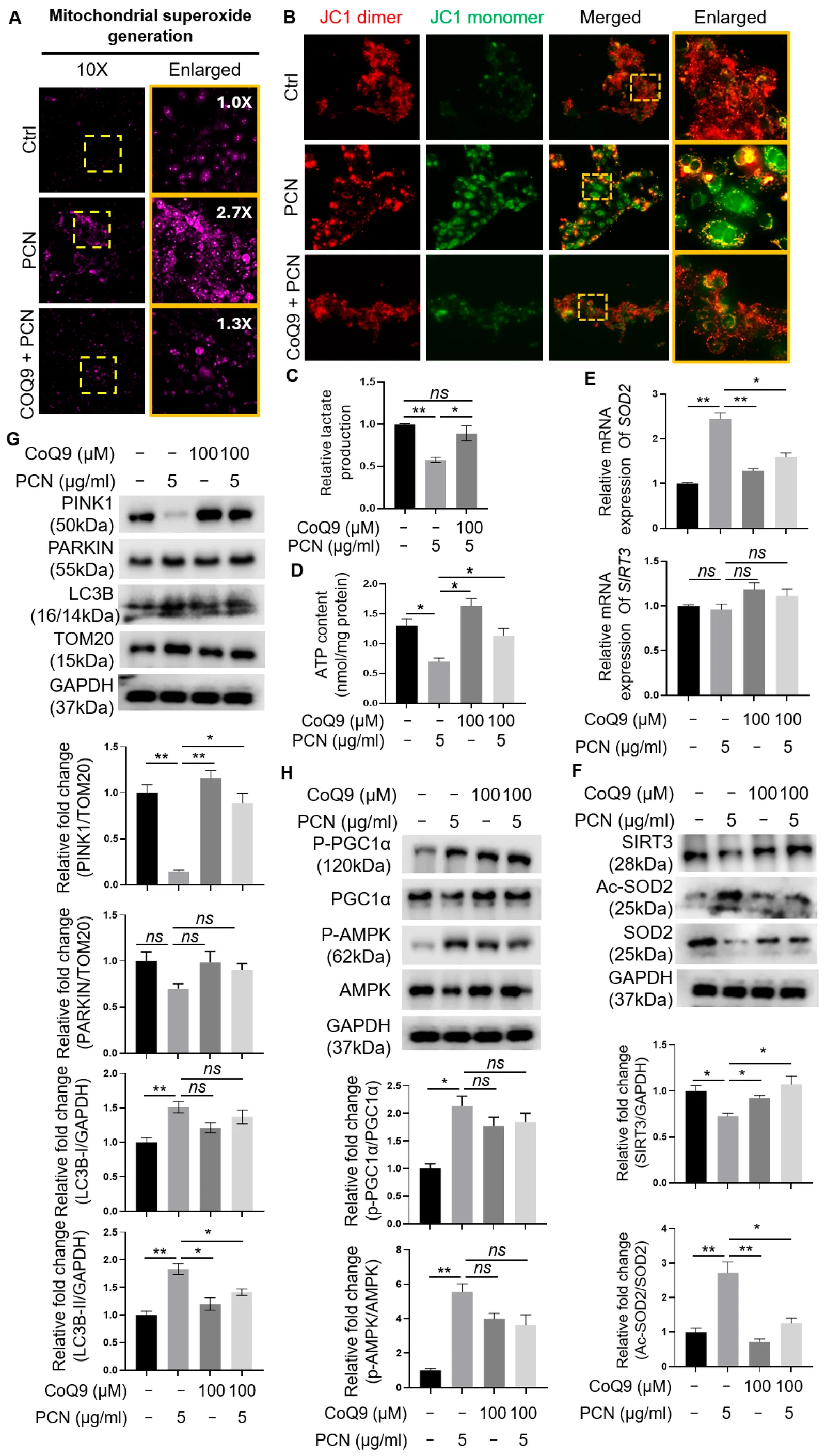
CoQ9 preserves mitochondrial integrity by regulating redox homeostasis, mitochondrial mitophagy, and biogenesis under PCN-mediated oxidative stress. (**A**) Submerged NHBE cells were pretreated with 100 µM CoQ9 for 1 h prior to 5 µg/mL PCN exposure for 6 h. Mitochondrial superoxide levels were visualized by incubation with MitoBright ROS Deep Red solution. Fluorescence intensities were quantified using ImageJ and normalized to the intensity of the untreated control group. Insets (yellow bold lines) correspond to the enlarged views of the indicated regions of interest (yellow dashed lines) (**B**–**F**) NHBE cells were pretreated with 100 µM CoQ9 for 1 h prior to 5 µg/mL PCN exposure for 24 h. (**B**) JC-1 mitochondrial membrane potential measurement. Submerged NHBE cells were exposed to PCN with and without CoQ9. JC-1 dimer and monomer were shown in red and green, respectively. Insets (yellow bold lines) correspond to the enlarged views of the indicated regions of interest (yellow dashed lines) (**C**) Lactate production levels were assessed in conditioned media from NHBE cells exposed to PCN and CoQ9. (**D**) Quantification of intracellular ATP levels. (**E**) Relative mRNA expression of *SOD2* and *SIRT3* measured by qPCR. (**F**) Western blot analysis of SIRT3, acetylated SOD2 (Ac-SOD2), and total SOD2 in whole cell lysates. (**G**) Western blot analysis of PINK, PARKIN, LC3B, and TOM20. (**H**) Western blot analysis of phosphorylated PGC-1α and AMPK, as well as total PGC-1α and AMPK in whole cell lysates. Immunoblot intensities were quantified by ImageJ and normalized to the untreated control group. Data are presented as mean ± SEM (n = 3). * *p* < 0.05, ** *p* < 0.01, *ns* = not significant by Student’s *t*-test.

**Figure 8:**
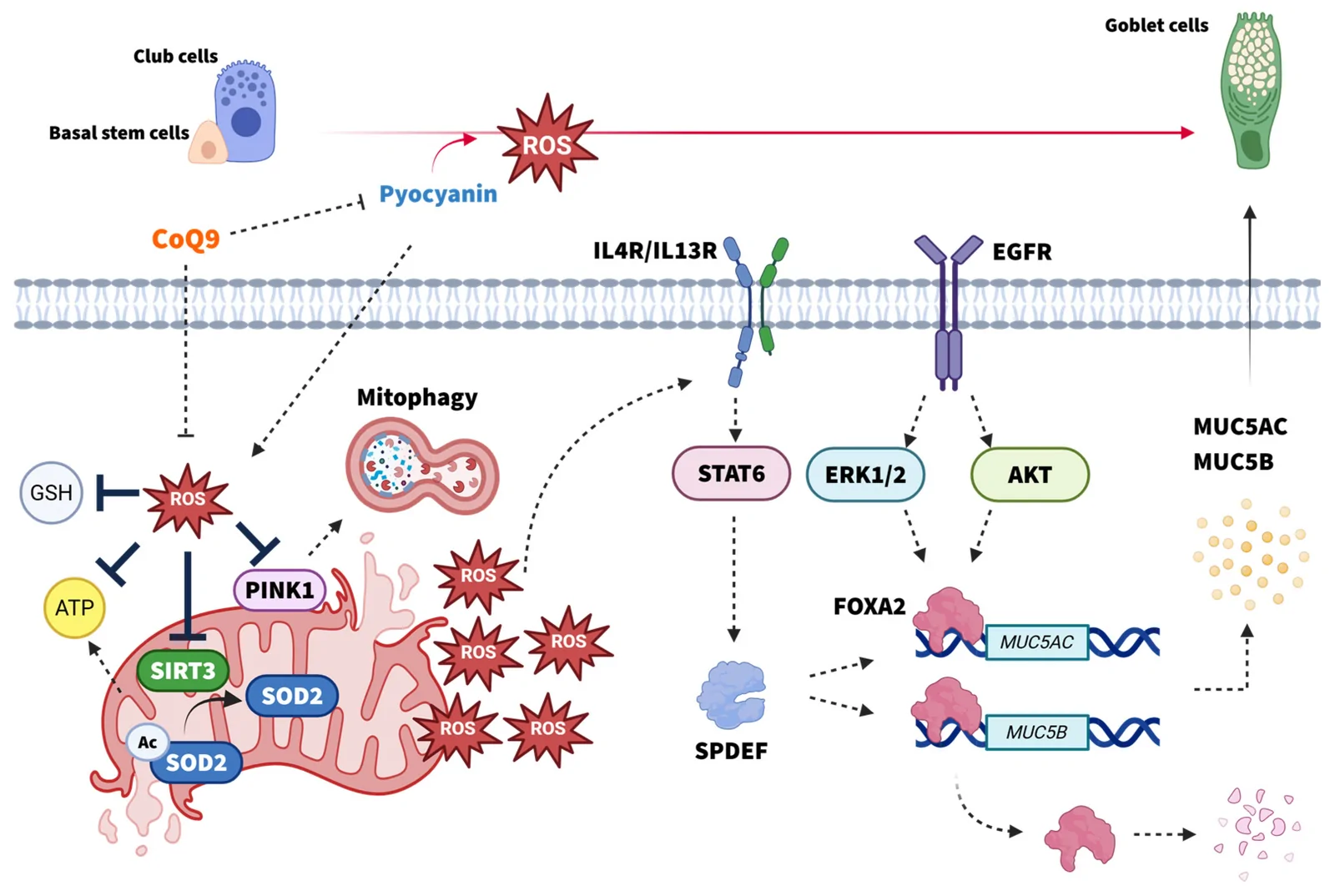
Schematics depicting the protective effects of CoQ9 on mucus pathology induced by PCN. CoQ9 reverses PCN-mediated goblet cell transdifferentiation from airway basal stem cells and club cells by inactivating mucinogenic signaling pathways, including EGFR-AKT/ERK1/2-FOXA2 and STAT6-SPDEF through downregulation of intracellular ROS levels. Antioxidant systems fail to scavenge PCN-induced intracellular ROS due to persistent reduction in GSH and accumulation of damaged mitochondria. CoQ9 restores redox homeostasis by improving GSH biosynthesis as well as mitochondrial integrity and dynamics under PCN exposure, which eventually downregulates mucin overproduction in bronchial epithelial cells.

## Data Availability

The original contributions presented in this study are included in the article/Supplementary Material. Further inquiries can be directed to the corresponding authors.

## Supplementary Materials

The following supporting information can be downloaded at: https://www.mdpi.com/article/10.3390/antiox15101225/s1, Supplementary Figures: Triplicate western blot analysis.
