# Supplementary material for: Coenzyme Q9 Derived from Oudemansiella raphanipes Attenuates Mucin Biosynthesis Induced by Pseudomonas aeruginosa Pyocyanin in Human Bronchial Epithelial Cells
Source: Antioxidants (Basel). 2026 Sep 23;15(10):1225. doi: 10.3390/antiox15101225 (PMC13700716; doi:10.3390/antiox15101225)
Supplement: Supplementary file 1 [file antioxidants-15-01225-s001.zip › antioxidants-4447805-Supplementary figures.pdf]

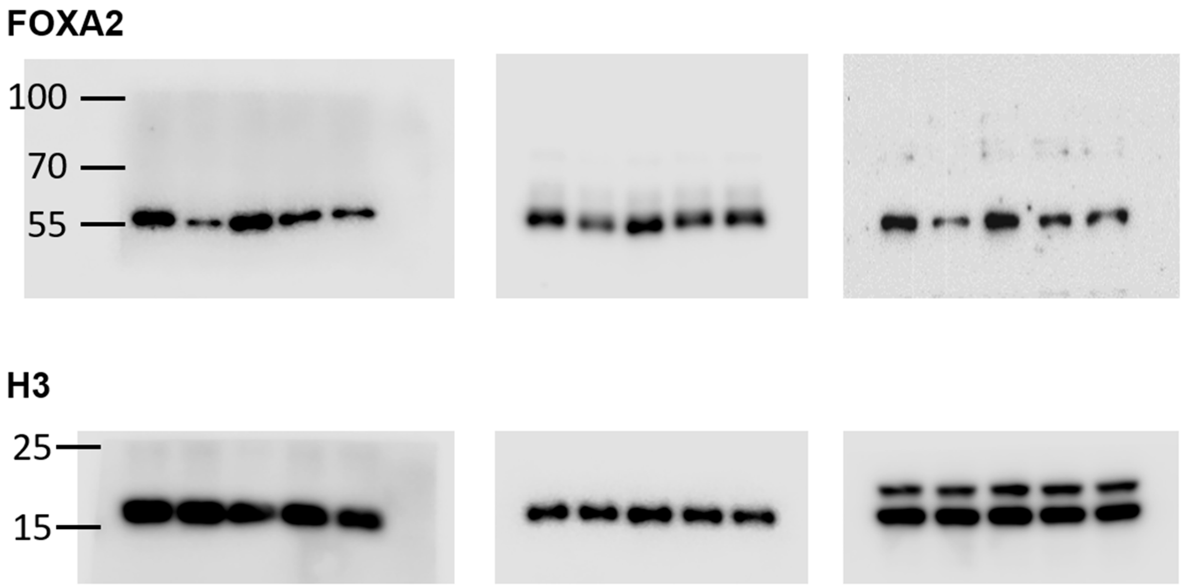

Figure S1. Triplicate western blot analysis of nuclear FOXA2 expression in co-enzyme Q9 (CoQ9)-pretreated and pyocyanin (PCN)-exposed primary normal human bronchial epithelial (NHBE) cells under submerged culture conditions.

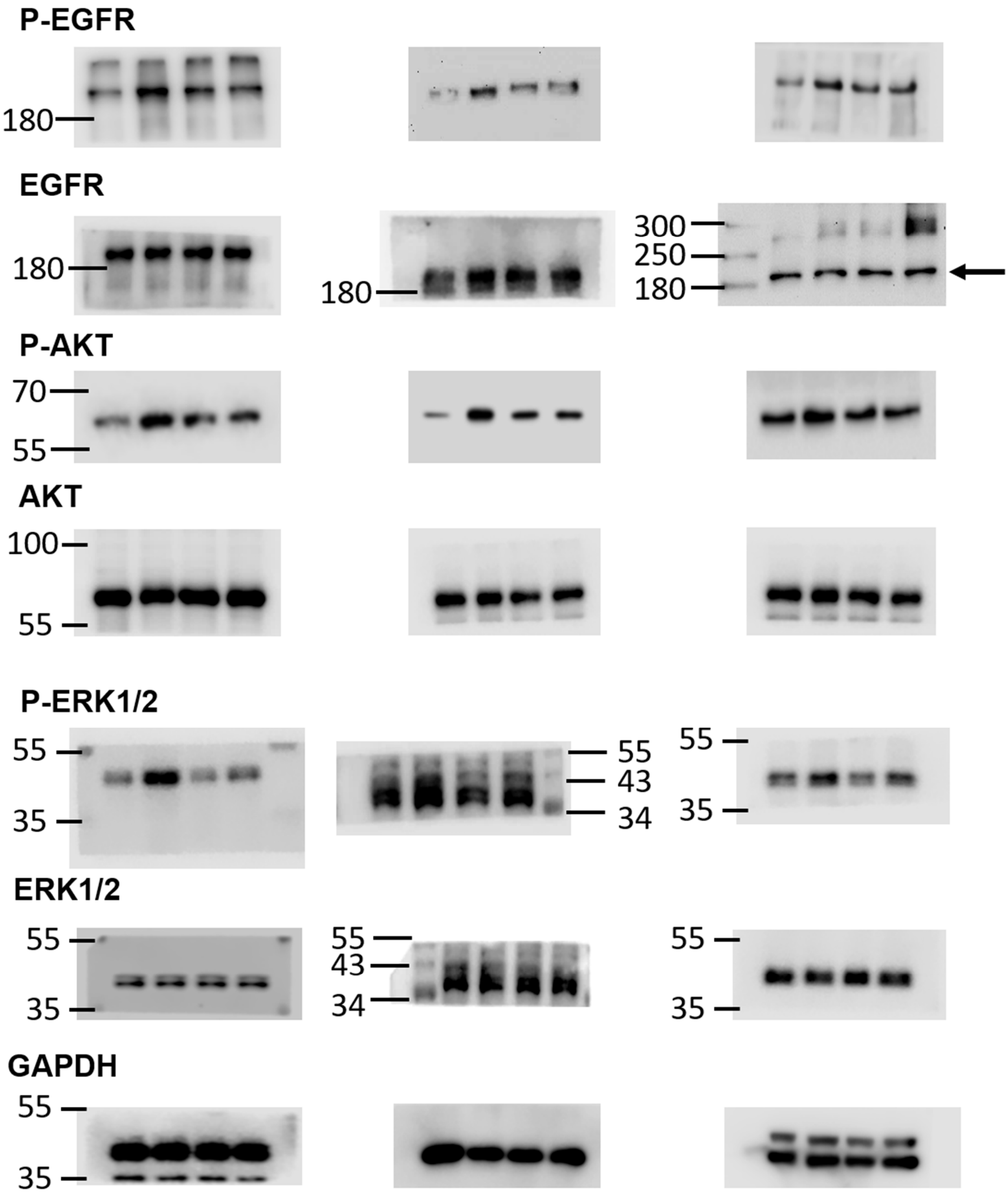

Figure S2. Triplicate western blot analysis of EGFR signaling cascades in CoQ9-pretreated and PCN-exposed NHBE cells

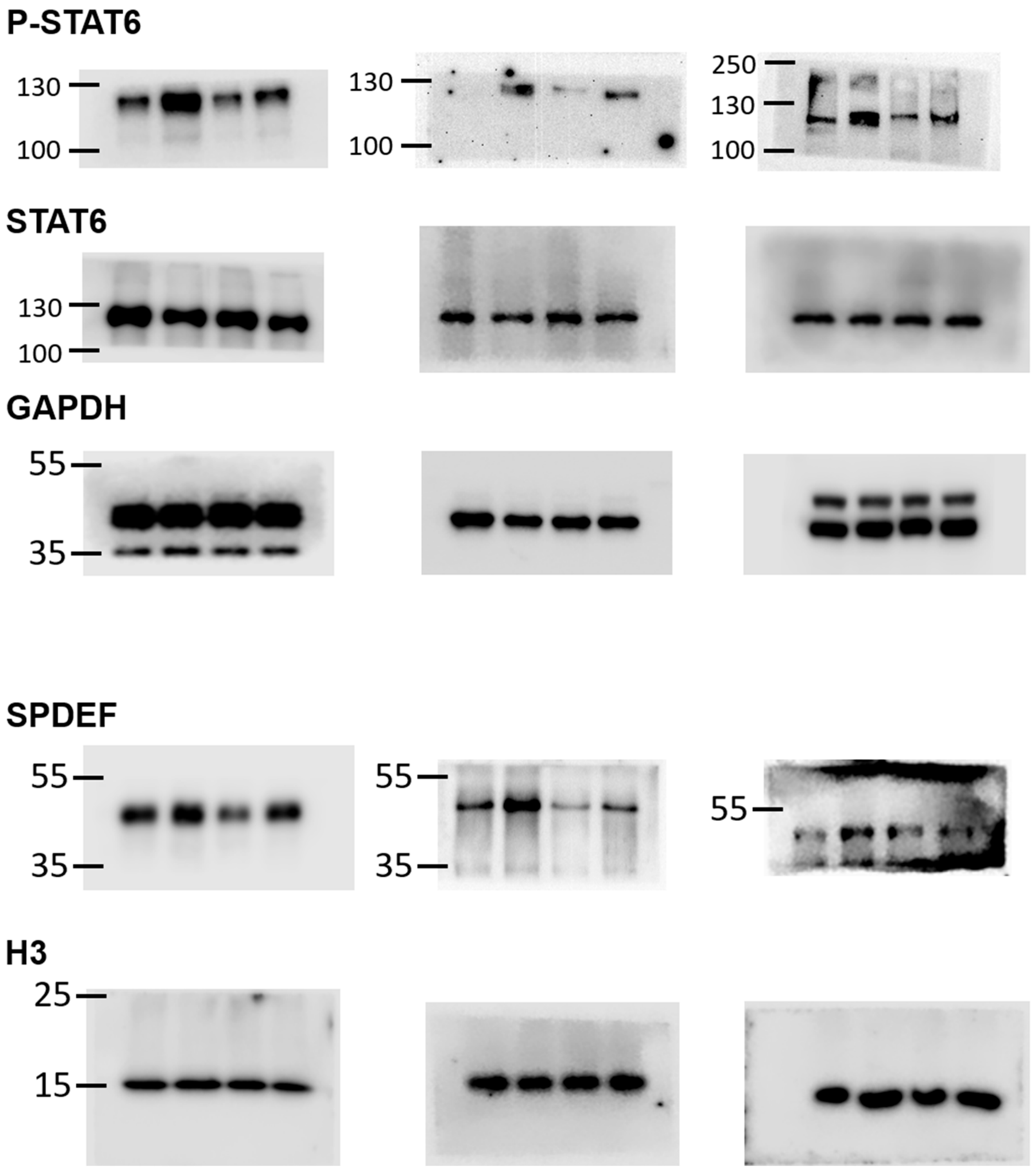

Figure S3. Triplicate western blot analysis of STAT6 signaling cascades in CoQ9-pretreated and PCN-exposed NHBE cells

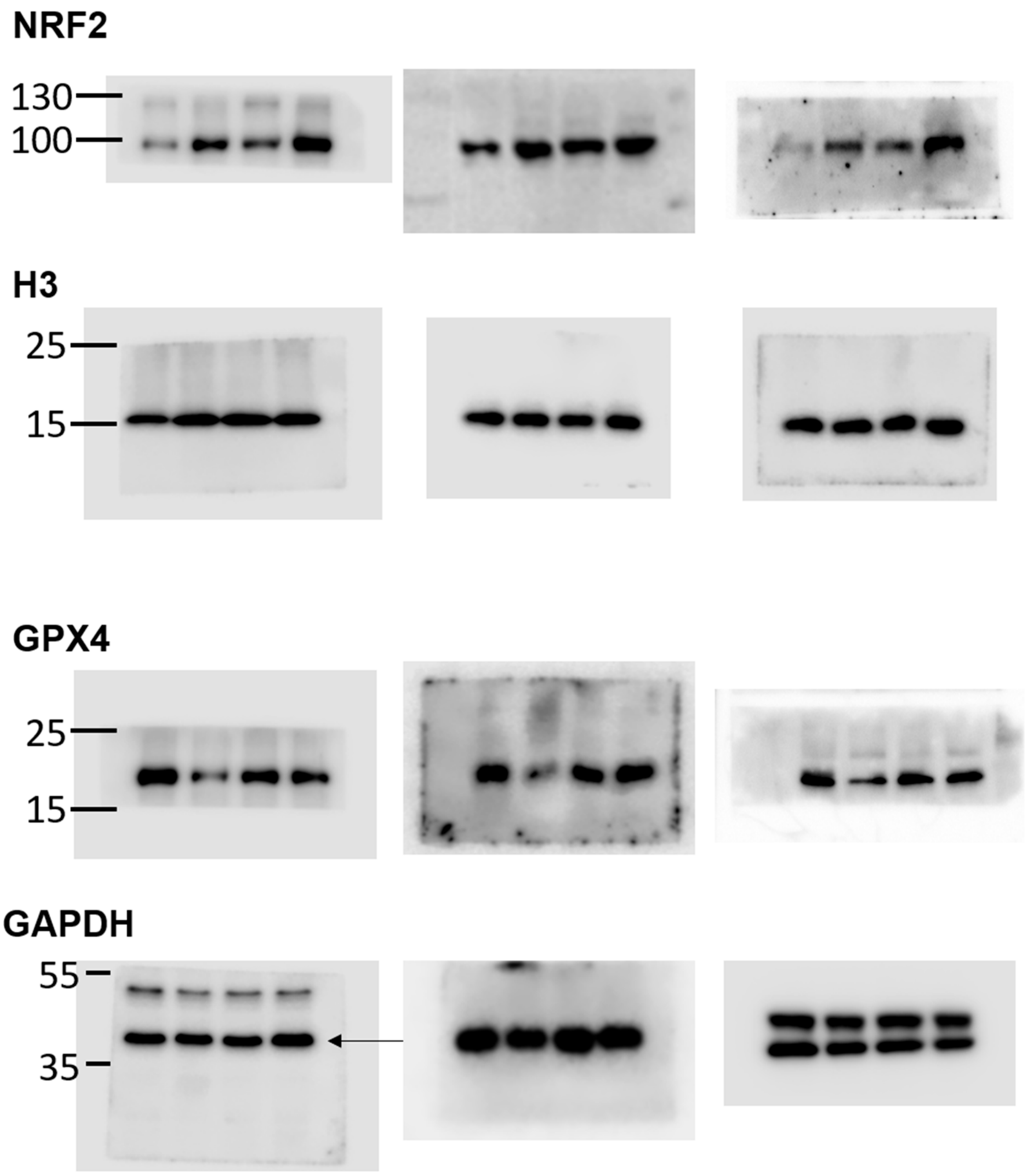

Figure S4. Triplicate western blot analysis of NRF2 and GPX4 expression in CoQ9-pretreated and PCN-exposed NHBE cells.

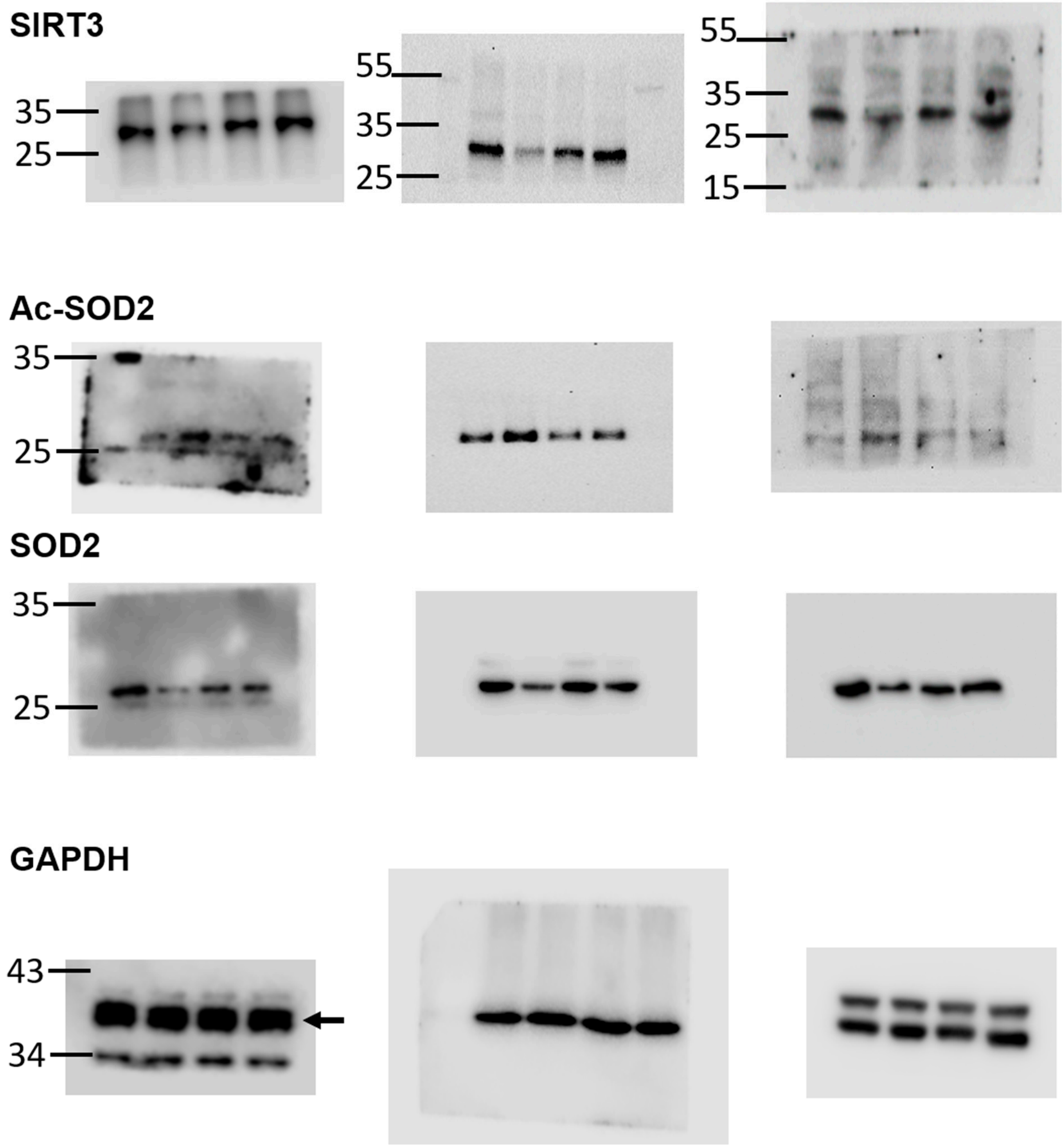

Figure S5. Triplicate western blot analysis of SIRT3, Ac-SOD2 and SOD2 expression in CoQ9-pretreated and PCN-exposed NHBE cells.

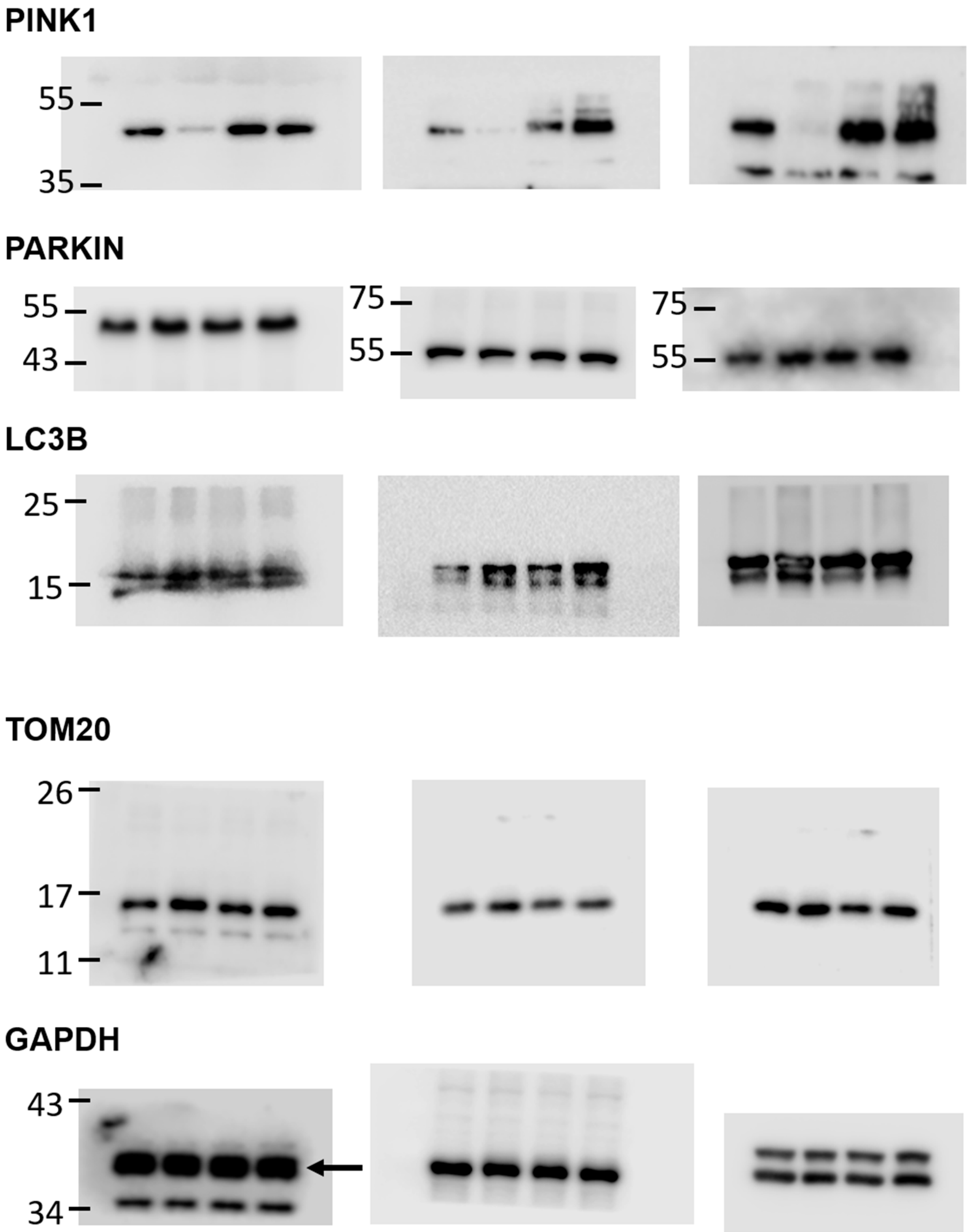

Figure S6. Triplicate western blot analysis of PINK, PARKIN, LC3B, and TOM20 expression in CoQ9-pretreated and PCN-exposed NHBE cells.

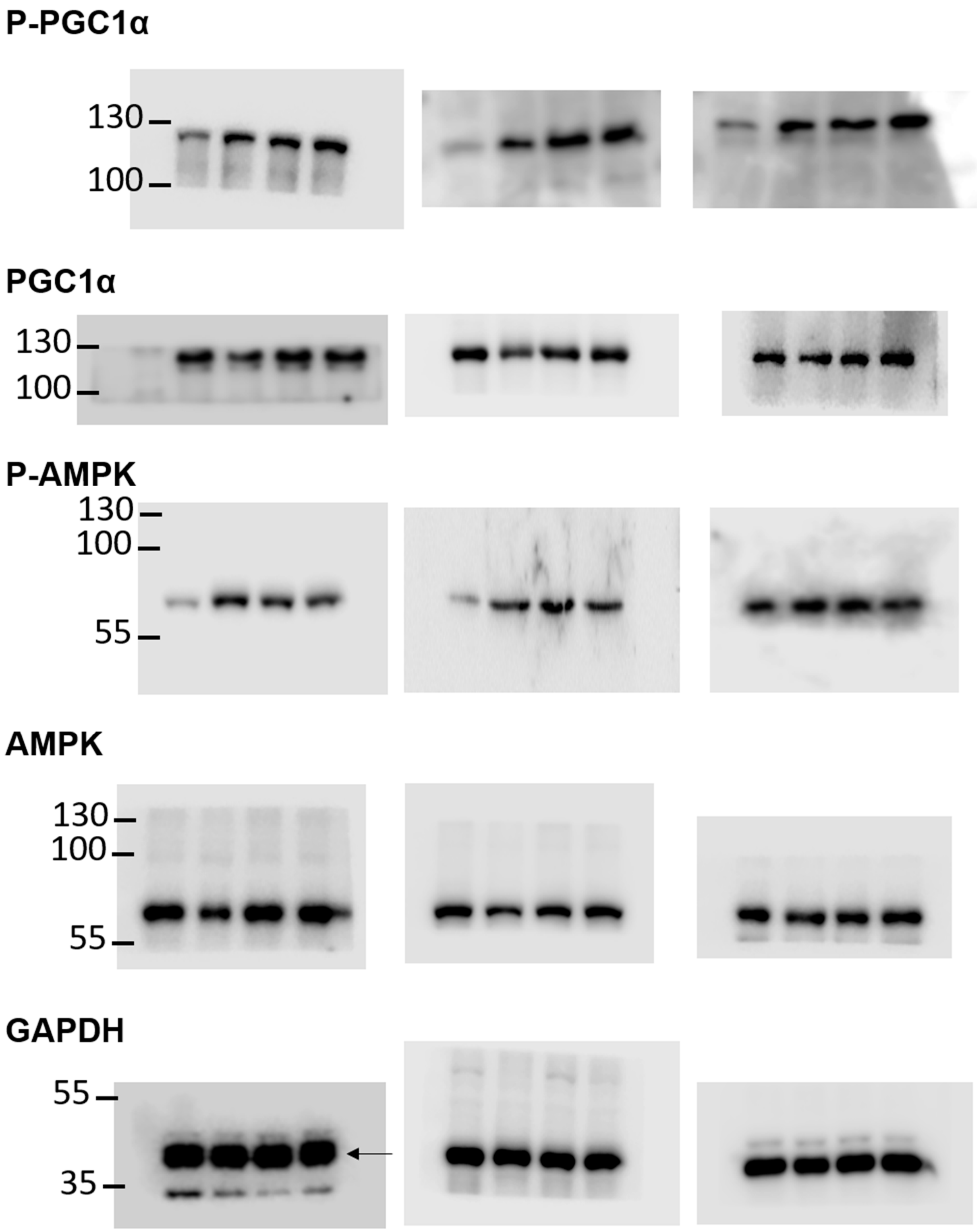

Figure S7. Triplicate western blot analysis of p-PGC-1α, PGC-1α, p-AMPK, AMPK by CoQ9 pretreatment in PCN-exposed NHBE cells.
